# *Tetraclinis articulata* (vahl) masters: An insight into its ethnobotany, phytochemistry, toxicity, biocide and therapeutic merits

**DOI:** 10.3389/fphar.2022.977726

**Published:** 2022-09-05

**Authors:** Sohaib Khatib, Mansour Sobeh, Latifa Bouissane

**Affiliations:** ^1^ Molecular Chemistry, Materials and Catalysis Laboratory, Faculty of Sciences and Technologies, Sultan Moulay Slimane University, Beni-Mellal, Morocco; ^2^ Agro Bio Sciences, Mohammed VI Polytechnic University, Ben‐Guerir, Morocco

**Keywords:** *Tetraclinis articulata* (Vahl) Masters, ethnopharmacology, biocide, phytochemistry, toxicity

## Abstract

*Tetraclinis articulata* (Vahl) Masters, commonly known as Sandarac tree and Araâr, is the only species representing the genus *Tetraclinis* Masters. The plant has been extensively used for medicinal, artistic, and ritual purposes since its first recorded use in 1800 B.C. Recently, a full range of ethnobotanical investigations has been undertaken to document the plant’s empirical knowledge. They reported the use of different parts, such as leaves, stems, cones, bark, and roots, as part of folk healing practices to manage diabetes mellitus, hypertension, fever, stomach disorders, and diarrhea, among others. The phytochemical studies have identified at least 130 compounds from leaves, cones, resin, bark, and woods. These chemical constituents are categorized into phenolic acids, flavonoids and their derivatives, volatile compounds, phytosterols, and fatty acids, among others. Furthermore, they have strongly been correlated with the promising antimicrobial, antioxidant, neuroprotective, antiurolithiatic, anti-inflammatory, antidiabetic, and cytotoxic properties of the plant. Toxicological studies argued that the plant is quite safe and devoid of eventual toxicity; however, in-depth investigations are required to validate the safety of the plant. The remarkable antimicrobial and antioxidant potencies of various extracts from the plant against a wide range of foodborne pathogens support their possible use to increase the shelf life of foodstuffs in the food industry. Likewise, various plant-based extracts have been proven to exert substantial biocidal properties, making them potential alternatives to synthetic pesticides in agriculture. The present review provides an up-to-date comprehensive insight about the ethnobotanical uses of *T. articulata*, along with its phytochemistry and biological activities to furnish directions for further studies. We also discussed the biocidal potency of the plant and highlighted its usage to extend the shelf life of perishable foods.

## Graphical Abstract

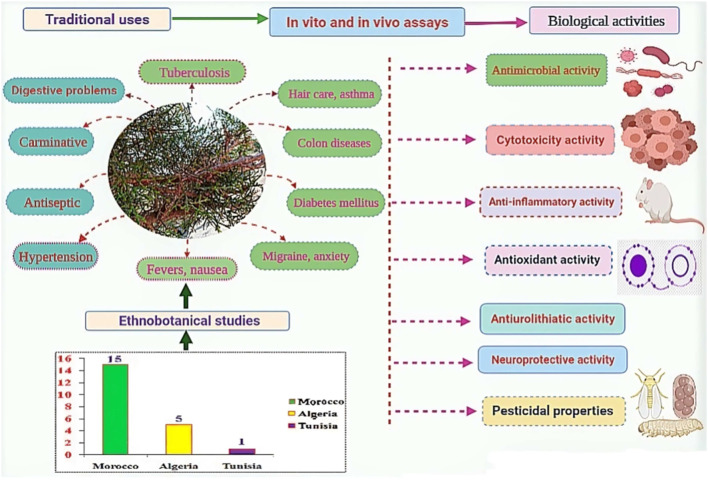

## Introduction

Since ancient times, medicinal and aromatic plants have been recognized as reliable sources of health-promoting bioactive agents and a valuable source for finding new drug leads (Kicel, 2020; Annaz et al., 2022). Recently, several serious concerns have been raised about the quality, efficacy, and safety of synthetic drugs (Sahoo et al., 2010). On the other hand, plant-based products are better for the environment and biologically sound as they are recognized by the cells of the body, allowing their metabolism to proceed (Van Wyk et Wink, 2015). As such, a plethora of medicinal and aromatic plants used in the traditional folk systems are progressively coming under the spotlight of scientific research to separate their active chemical ingredients for use in modern dispensing formulas (El-Hossary et al., 2020; Khatib et al., 2022).

The use of *T. articulata* dates back to Phoenician and Roman times. Due to its high flammability, the wood was used as a source of fuel (firewood and charcoal), incense sticks in religious ceremonies, and as an embalming material. It has been also used as planks in mining, while exquisite wood carvings have been made and exported to Mediterranean countries (Farjon, 2010; Morte and Honrubia, 1996). Nowadays, *T. articulata* covers an area of 566.000 ha in Morocco, categorized into six major distinct biogeographic zones, namely the Rif, eastern Middle Atlas and oriental, Western Middle Atlas, High Atlas, valleys of the central and the western plateau (Sadiki et al., 2018). However, *T. articulata’*s total area is constantly shrinking year by year due to the overbrowsing (especially by goats) (Kahouadji et al., 2022), making all attempts to preserve this plant and ensure its sustainability utterly critical (Bouhtoury-Charrier et al., 2009). As such, it is enlisted in the red list of IUCN of threatened conifer, and numerous countries have enacted legislation to ensure its protection (Sánchez-Gómez et al., 2013). Gum sandarac is the name given to the resin produced by this plant, which is released spontaneously in the form of nodules or retrieved by incisions made in the tree bark. The resin exudates are highly prized in the manufacture of varnishes, dental fillings, and pounce (Buhagiar et al., 2000). The tree resin also has a myriad of industrial applications, including replacing Canadian balsam in the preparation of microscope slides (www.conifers.org/cu/Tetraclinis.php, accessed on 18 November 2021). Essential oil derived from the tree resin is almost colorless or pale yellow with a slightly balsamic aroma applied as a fixative, relaxant, and treatment for stress relief and cold (El Moussaouiti et al., 2010). Wood derived from roots and logs is widely known as thuya wood or citron wood. The attractive burled root wood is potent and shines like glass when polished, making it a popular choice to fabricate one-of-a-kind and exquisitely handcrafted goods, such as tables, lamps, jewelry boxes, mirrors, trays, desktops items, and pens, among others (Buhagiar et al., 2000; El Moussaouiti et al., 2010). In North Africa, various parts of this tree, such as leaves, stems, roots, fruit, and seeds, have been used in the traditional folk medicine against multiple health conditions. Different parts of the plant have been reportedly prepared in the form of decoction, infusion, fumigation, and paste and applied topically or orally to treat diabetes mellitus, hypertension, diarrhea, rheumatism, intestinal, respiratory, and skin diseases (Kachmar et al., 2021; Idm’hand et al., 2020; Amel, 2013; Buhagiar et al., 2000). Recent studies have lent credence to several ethnomedicinal applications of *T. articulata* including, antioxidant (Djouahri et al., 2016), antimicrobial (Bahri et al., 2015), anti-inflammatory (Rached et al., 2018), neuroprotective (El Jemli et al., 2016; Sadiki et al., 2018b), vasorelaxant (Zidane et al., 2014), and anticancer properties (Calderón-Montaño et al., 2021). They also identified a wide range of bioactive compounds belonging to phenolic acids, flavonoids and their derivatives, fatty acids, terpenes, and phytosterols (Djouahri et al., 2014; Bahri et al., 2015; Rached et al., 2018).

Herein, we aimed at providing an up-to-date comprehensive overview of the ethnomedicinal uses of *T. articulata* (Vahl) Masters in the North Africa region, where this species thrives based on plenty of ethnobotanical surveys. The phytochemistry and biological activities of this tree are also critically summarized and reported in our review. We also discussed for the first time the ethnoveterinary applications and biopesticidal potential of *T. articulata* and the possibility of its application in agriculture to achieve pest management in an eco-friendly way. Moreover, we reported its-related patents published between 2001 and 2022 and highlighted the use of *T. articulata* extracts as a food preservative to extend the shelf life of perishable foodstuffs in the food industry.

## Methods

**FIGURE 1:**
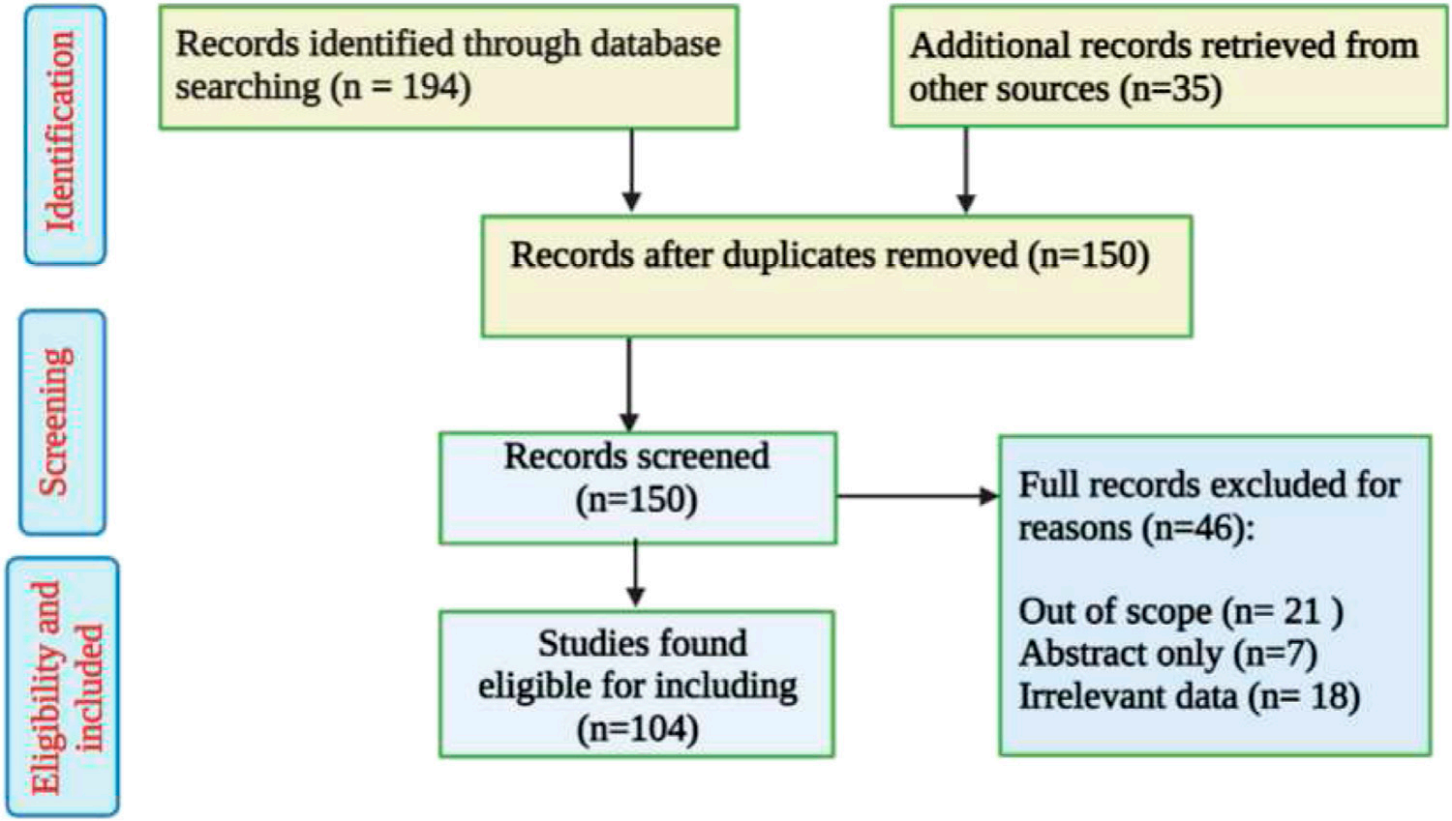
Flowchart of *T. articulata* studies inclusion and exclusion criteria.

An extensive literature-based search was performed using different databases such as ScienceDirect, PubMed, Google Scholar, Scopus, Springer, and SciFinder. Only peer-reviewed publications were retrieved using the search key phrase ‘*Tetraclinis articulata*’ with no time limitation set. Duplicated and irrelevant works were excluded (Figure 1). Additionally, numerous books with botanical and ethnopharmacological material were also reviewed**.** Furthermore, the online web server ProTox-II (http://tox.charite.de/protox_II, accessed on 26 April 2022) was used to predict the organ toxicity and some toxicological endpoints of phenolic compounds, as well as the predominant volatile compounds, detected in *T. articulata* (Banerjee et al., 2018). The species name and its synonyms were checked based on the online database (http://www.worldfloraonline.org).

## Botanical characteristics of *T. articulata* (Vahl) masters

### Taxonomy

*Tetraclinis articulata* (Vahl) Masters, commonly known as Sandarac tree, and Araâr (Table 1), is a monoïc species that blooms in the spring with long-lasting foliage (Djouahri et al., 2014). Its taxonomy has long been a matter of debate; it was included in the Callitroideae Subfamily (genus Callitris Vent.), which has been around for a great many years. However, *T. articulata* is unique in this subfamily since all other species are found only in the southern hemisphere, whereas *Tetraclinis* spreads across the Mediterranean. It was also once classified as part of the genus *Thuja* L. and *Widdringtonia* Endl (Jagel and Stützel, 2003; Sánchez-Gómez et al., 2013). Recent morphological and molecular studies confirmed the occurrence of *T. articulata* within the Cupressaceae family as the only species of the genus *Tetraclinis* Masters (Jagel and Stützel, 2003; Sánchez-Gómez et al., 2013; Jlizi et al., 2018). The common names and synonyms of the tree have been collected and listed in Table 1.

**TABLE 1:** Synonyms and common names of *T. articulata* (Vahl) Masters according to the World Flora Online (http://www.worldfloraonline.org/taxon/wfo-0000456325. Accessed on: 03 August 2022).

Synonyms*	References	
*Callitris articulata* H.Karst	http://www.worldfloraonline.org/taxon/wfo-0000456325	
*Callitris macrostachya* Steud				
*Callitris quadrivalvis* Rich. and A.Rich				
*Callitris triquetra* Loudon				
*Cupressus articulata* (Vahl) J.Forbes	*Confidence level: III (High Confidence level)	
*Cupressus triquetra* Lodd. ex Loudon				
*Cupressus triquetra* Jacques				
*Juniperus cunninghamii* Carrière				
*Thuja articulata* Vahl				
**Common names**		
Arabic	*Araâr, Berbouch, Chajarat Al-Hayat, Megloub, Dbagha*	Buhagiar et al., 2000
		Miara et al., 2019
Amazigh	*Azuka*	Buhagiar et al., 2000
French	*Thuya de Barbarie, Bois de Citre, Thuya d’Algérie*	Farjon, (2010)
English	*Sandarac Gum-Tree, Cartagena Cypress, Arar*	Buhagiar et al., 2000
Spanish	*Sabina mora, Alerce, Tuya articulada*	Farjon, (2010)

### Botanical description

**FIGURE 2:**
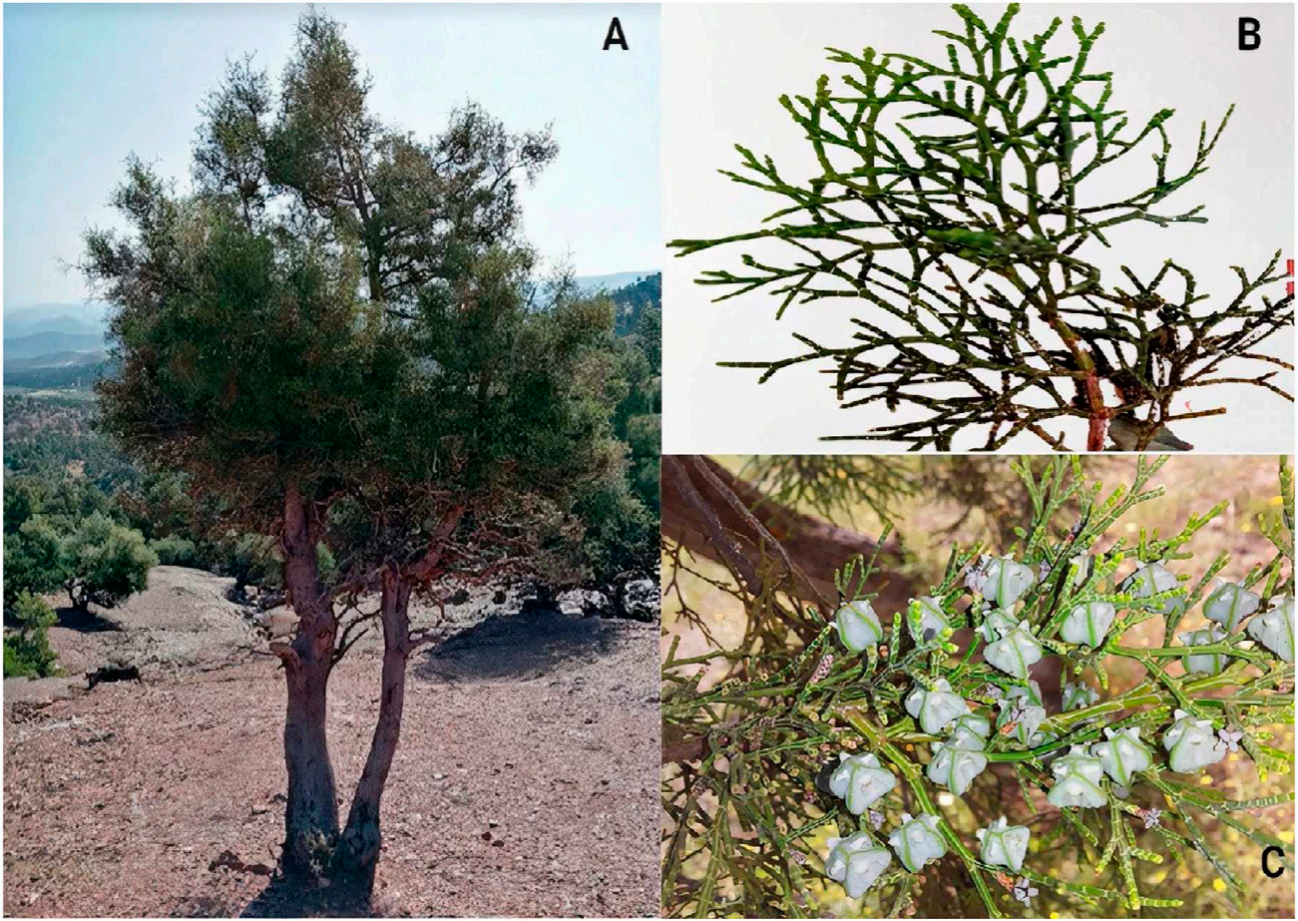
Different parts of *T. articulata* (Vahl) Masters **(A)** the whole plant; **(B)** Leaves; **(C)** Fruits (Pictures were taken in the region of Beni Mellal-khenifra, Morocco, 32⁰54′26.9″N 6⁰16′51.5″W) 2022^©^.

*T. articulata*, widely known as Sandarac tree and Araâr (Table 1), is an evergreen, monoecious tree belonging to the family of Cupressaceae. It is a slow-growing medium-sized tree reaching 6–8 (rarely 15) m in height; the monopodial or multi-stemmed trunk is reddish-browns with vertical stripes and sweet-smelling, generally low branching, up to 50 cm diameter, converging from the base. The root system is profound and not very densely ramified. Young branches are bright green, flattened, flexible, and articulated. Many of these branches fall during the dry season, giving the species greater chances of surviving the long, and harsh summer. The leaves are grouped in opposite decussate pairs, with successive pairs closely and then gradually distanced, appearing in whorls of four on slender branchlets. The young leaves (appearing in the first year) are glaucous, needle-shaped, and about 5–10 cm long (the true leaves appear from the second year onwards). The flowers are monoecious. The male flowers are on the ends of the branches, and the female flowers are on the sides. The ovules are upright, bottle-shaped, and visible. The cone is ovoid, subglobose, with a diameter of approximately 8–12 mm. At first, it is glaucous, tetragonal, and then becomes light brown within 1 year from pollination with thick four woody scales placed in two opposite pairs**.** Two of the scales are truncated with deep, vertical depression, and the other two are sharp and wide. Seeds (34 × 11.5 mm) are elongated, with resinous peripheral sacs, and have two membranous wings up to 8 x 4-5 mm in size in the form of a samara. There are three–six cotyledons. The number of chromosomes is 2n = 22, (Figure 2) (Morte and Honrubia, 1996; Buhagiar et al., 2000; Farjon, 2010).

### Geographical distribution

**FIGURE 3:**
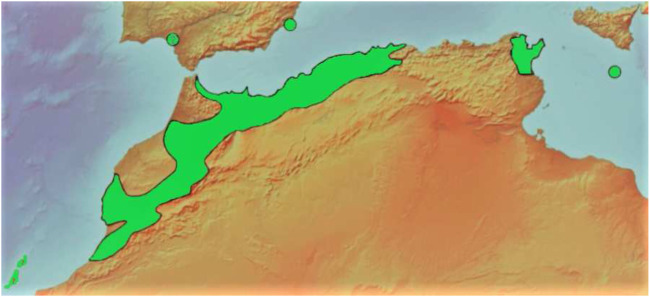
Geographical distribution of *T. articulata*, 2022^©^.

This evergreen coniferous tree is endemic to northwestern Africa in the Atlas Mountains of Morocco, Algeria, and Tunisia, with two small outlying populations in Malta and east Spain (nearby Cartagena and the province of Murcia) (Figure 3) (Bouhtoury-Charrier et al., 2009; Achmit et al., 2021). It is worth noting that the Sierra de Cartagena (Sierra de Cartagena) in east Spain is considered the only natural stronghold of the species in Europe (Morte and Honrubia, 1996). There are also scattered localities deemed naturalized or of unclear origin in the Canary Islands, Cyprus, and the southern Spanish provinces of Málaga (Monte San Antón), Granada (Barranco de Lanjarón), and Huelva (Doñana) (García-Castaño et al., 2021). Their occurrence might be accounted for by the abrupt climatic changes that have taken place in Mediterranean regions over the previous millennia, which may have permitted the import of a variety of plant species from North Africa (Morte and Honrubia, 1996).

### Edaphic and pedoclimatic conditions

This thermophilous and xerophytic species thrives in a hot, dry subtropical Mediterranean climate at relatively low altitudes (El Moussaouiti et al., 2010). *T. articulata* grows from sea level up to 1,000-1,100 m on shady slopes and 1,500-1,600 m in sunny places (Morte and Honrubia, 1996; Sánchez-Gómez et al., 2013). Nonetheless, it has been spotted in Spain at altitudes beneath 370 m, where there is no threat of frost. *T. articulata* commonly emerges from tree stumps and can withstand wildfires and moderate levels of animals grazing due to its coppicing ability (El Moussaouiti et al., 2010). It grows mainly in calcareous soils, but it can acclimate to other soil types, such as limestone, dolomite, rhyolites, granite, and schist, except for sand-filled environments (Morte and Honrubia, 1996; Sánchez-Gómez et al., 2013). It also appears to stand up to drought (less than 250 mm of rainfall per year) and heavy metals like Zn and Pb in soils, rendering it a strong candidate for infertile and polluted land rehabilitation (Morte and Honrubia, 1996; Buhagiar et al., 2000; Sánchez-Gómez et al., 2013).

## Ethnobotanical aspects of *T. articulata* (vahl) masters

### Ethnomedicinal uses in humans

**FIGURE 4:**
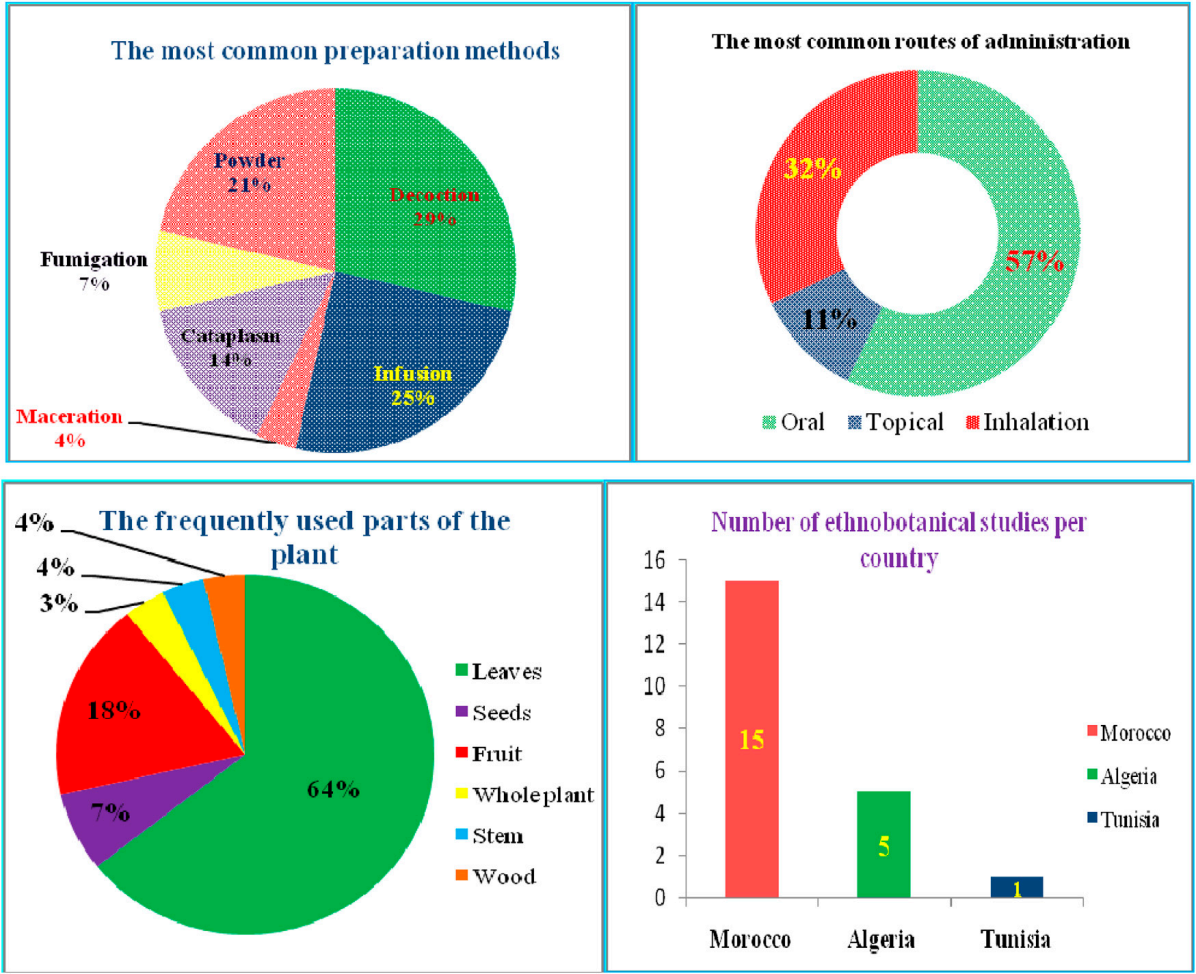
The most common preparation methods, routes of administration and used parts of *T. articulata,* as well as the number of ethnobotanical studies in each country.

According to our thorough literature search, 21 ethnobotanical studies carried out in the North Africa region mentioned the ethnomedicinal and ethnoveterinary uses of *T. articulata*. These surveys were especially undertaken in Morocco with 15 ethnobotanical investigations (Figure 4). They reported that various parts of this tree, such as leaves, seeds, fruit, stems, have been used to prevent and treat multiple pathological health conditions. The plant’s aqueous extracts prepared as a decoction (29%), infusion (25%), maceration (4%), as well as cataplasm (14%), and fumigation (7%) were the predominantly reported mode of preparation; whereas, stomach pain, respiratory and intestinal infections, diabetes, and hypertension were the frequently treated diseases (Figure 4; Table 2). Merzouki et al. (2000) reported that aboriginal communities in the Ksar Lekbir district of Morocco used the leaf or fruit infusion orally as digestive, carminative, and for cough and asthma treatment. For hair care, they blended the powdered leaves of *T. articulata* with those of *Lawsonia inermis* L, added water, and applied the paste topically to their hair. To manage diabetes mellitus, Laadim et al. (2017) stated the local communities in northwestern Morocco drank leaf infusion over a period varying from 1 week to 1 month. This claim has been corroborated by several ethnobotanical investigations, such as the one conducted in the Central Middle Atlas region of Morocco by Hachi et al. (2016) and also by another one carried out by Rachid et al. (2012) in Algeria, wherein indigenous people took the leaves’ maceration to treat diabetes mellitus. In addition, the leaves or fruits of this plant are prepared as an infusion, decoction, or poultice and used topically or orally to treat digestive, urinary, circulatory, and respiratory disorders as well as skeleton and nervous problems (Ouhaddou et al., 2014). The skimmed milk combined with the leaves and cones decoction has been used as an expectorant and emetic to treat intoxication, diarrhea, and stomach pain (http://temperate.theferns.info/plant/Tetraclinis+articulata, 2021). Besides, the decoction of the leaf is believed to be beneficial against bruises and wounds when applied topically, whereas the macerated leaves serve to make tea (M’barek et al., 2018). Burnt resin is used as evening incense; its balsamic-like scent has been believed to have a relaxing and calming effect. It has also been said to be beneficial in cases of insomnia caused by stress (http://temperate.theferns.info/plant/Tetraclinis+articulata, 2021). In-depth details about the ethnobotanical uses of *T. articulata*, mode of preparations, routes of administration, and parts used were gathered and listed in Table 2. The figures below are based on 21 ethnobotanical studies conducted exclusively in the North Africa region.

**TABLE 2:** Sum-up of the ethnobotanical uses of *T. articulata.*

Vernacular names	Parts used	Ethno-preparations	Mode of administration	Ethnobotanical uses	References
**Algeria**					
*Araâr*	Nr	Fumigation	Nr	Antiseptic	Nazim et al. (2020)
*Araâr*	Le, Sd	Decoction	Oral, inhalation	Cough, flu	Zatout et al. (2021)
*Araâr*	Nr	Nr	Oral, topical	Respiratory tract and intestinal infections. Externally, it is used to scar the umbilical wounds of neonates	Belgacem et al. (2021)
*Araâr*	Le, Fr, AP	Infusion, Decoction, Cataplasm	Oral, topical	Tuberculosis, stomach disorders, diarrhea, cough, infection of the urinary tract, nausea, migraine, anxiety, and colon diseases	Senouci et al. (2019)
*Araâr*	Le	Maceration	Oral	The leaves’ maceration is taken to treat diabetes mellitus	Rachid et al. (2012)
**Morocco**					
Araâr	Le, St	Decoction	Oral	Antidiarrhea, hair care, digestive diseases, and as a natural vomiting agent	Jaadan et al. (2020)
Azuka	Le	Decoction, Powder	Oral	Digestive disorders, gastric folds, abdominal pain and diarrhea	Sbai-Jouilil et al. (2017)
*Araâr*	Le	Infusion	Oral	To treat diabetes mellitus, an infusion of the plant’s leaves is taken orally for a period of 1 week to 1 month	Laadim et al. (2017)
Azouka, Aârar	Le, Fr	Decoction, Powder, Infusion, Cataplasm	Oral, topical	Digestive, urinary, and respiratory disorders, circulatory, skeleton, and nervous problems	Ouhaddou et al. (2014)
Azouka, *Araâr*	Le, Fr	Infusion	Oral	Carminative, asthma, cough, and digestive problems	Abouri et al. (2012)
*Araâr*	Le	Powder, Cataplasm	Topical, oral	Poultice leaves are applied to the head to treat fevers, while powdered leaves are used to treat abdominal pains and colds	Fadili et al. (2017)
*Araâr*	Le, Br	Decoction	Oral	Diabetes Mellitus	Hachi et al. (2016)
*Araâr*	Le	Powder	Oral	Diabetes Mellitus	Idm’hand et al. (2020)
*Araâr*	Sd, Le	Nr	Oral	Diabetes Mellitus, Hypertension	Amel, (2013)
*Araâr*	Le, WP, Fr	Decoction, Powder, Infusion	Topical, inhalation	Leaves, fruits, and the whole plant are crushed to obtain a powder, or subjected to decoction/infusion, then mixed with honey and alum and applied topically to treat rheumatism, foot, sciatic nerve, sprains, burns, pimples, and skin problems	Fakchich and Elachouri, (2014)
*Araâr*	Le, Fr	Infusion	Topical, oral	The infusion of leaves and fruits has been used as a carminative and to cure coughs, asthma, and digestive problems. The leaves of the plant are mixed with those of *Lawsonia inermis* L. powdered and blended with water and applied as a hair tonic. Also, the resin has been used as incense in ceremonial rites	Merzouki et al. (2000)
*Araâr*	Le	Cataplasm	Topical	Dermatological diseases such as eczema	Chaachouay et al. (2021)
*Araâr*	Le	Decoction	Oral	Leaves decoction is blended with milk and used by local people as an analgesic agent, to treat rheumatic and stomach disorders	Khabbach et al. (2012)
*Araâr*	Le	Infusion, Fumigation	Oral, inhalation	Hypotensive agent, diabetes mellitus, and stomachache	Kachmar et al. (2021)
*Araâr*	Le	Powder	Topical	The leaves powder is sprinkled immediately on the burns	Salhi et al. (2019)
Araâr	Le	Powder	Topical	The leaves powder is sprinkled immediately on the burns	Mrabti et al. (2022)
*Araâr*	Le	Powder	Oral	Antihypertensive use	Idm’hand et al. (2022)
**Tunisia**					
*Araâr*	Wd	Tar (The plant’s wood distillation)	Topical	A distilled dried wood-based cream is applied topically to treat parasitic diseases, inflamed wounds, and scabies in camels	Viegi and Ghedira, (2014)

AbbreviationsLe, Leaves; Fr, Fruits; WP, whole plant, Sd, Seeds; St, Stem; AP, aerial parts; Br, Branch; Nr, Not reported.

### Ethnoveterinary uses

Ethnoveterinary medicine is a traditional multifaceted system of concepts, methods, skills, and practices, which have been applied to prevent, cure, and maintain livestock health (McGaw and Eloff, 2008). It mainly relies on plant-based remedies for the treatment and prevention of microbial diseases and parasites in cattle. The ethnoveterinary practices are widespread in the areas where indigenous medical knowledge is deeply ingrained (Abdalla and McGaw, 2020). During the last decades, the overexploitation of standard veterinary drugs has led to severe hazardous effects on both humans and animals, including allergic reactions in hypersensitive subjects, as well as the emergence of resistant bacteria strains, rendering a plethora of these veterinary medications ineffective. Furthermore, the majority of livestock farmers in poor-resource countries neither have easy access to veterinary drugs nor can afford the costs (Lans et al., 2007). On the other hand, ethnoveterinary practices are inexpensive because they rely on readily available local sources. Hence, if appropriately harnessed, they may serve as a suitable alternative to modern veterinary medications for livestock care (Abdalla and McGaw, 2020). They also remain a choice even for rich livestock raisers, especially if animal-related veterinary charges are expensive, and the animal’s market value does not warrant the cost of modern veterinary care (Lans et al., 2007). Although these practices have been handed down orally over generations, recent ethnoveterinary surveys indicated that the know-how related to livestock healthcare is mainly held by elderly people, particularly men who are often in charge of herds (McGaw and Eloff, 2008; Ul Hassan et al., 2014; Piluzza et al., 2015; Miara et al., 2019). Thereby, these practices are likely to vanish along with their owners. In North Africa, many efforts have been invested into documenting and preserving ethnoveterinary knowledge in order to ensure its sustainability for the forthcoming generations. In Tunisia, Virgo and Ghedira, (2014) conducted an ethnoveterinary study to collect data about medicinal plants used locally to treat animals. They reported that indigenous people applied tar (a liquid obtained through destructive distillation or carbonization of dried wood)-based cream of *T. articulata* topically to treat parasitic diseases, scabies, and inflamed wounds in camels.

To manage several diseases in sheep, Miara et al. (2019) stated that indigenous nomadic people wildcrafted the aerial parts of this plant to prepare an infusion or decoction, which is then fed to livestock to cure stomach disorders, diarrhea, kidney problems, and tremor. In Morocco, herders and farmers hold advanced ethnoveterinary knowledge; they used their medical skills not only to treat animals but also to improve the quality of their herds’ milk, dairy products, and meat. They utilized dried wood tar of *T. articulata* to treat skin and dermal diseases, including inflamed wounds, scabies, and internal parasites (Abdalla and McGaw, 2020). Similarly, a recent study carried out in the Rif area, Northern Morocco, by Chaachouay et al. (2022) reported that the native people heavily rely on the crushed leaves from this species to heal goats’ Endo- and ectoparasite infections.

## Chemical composition

### Essential oil

**FIGURE 5:**
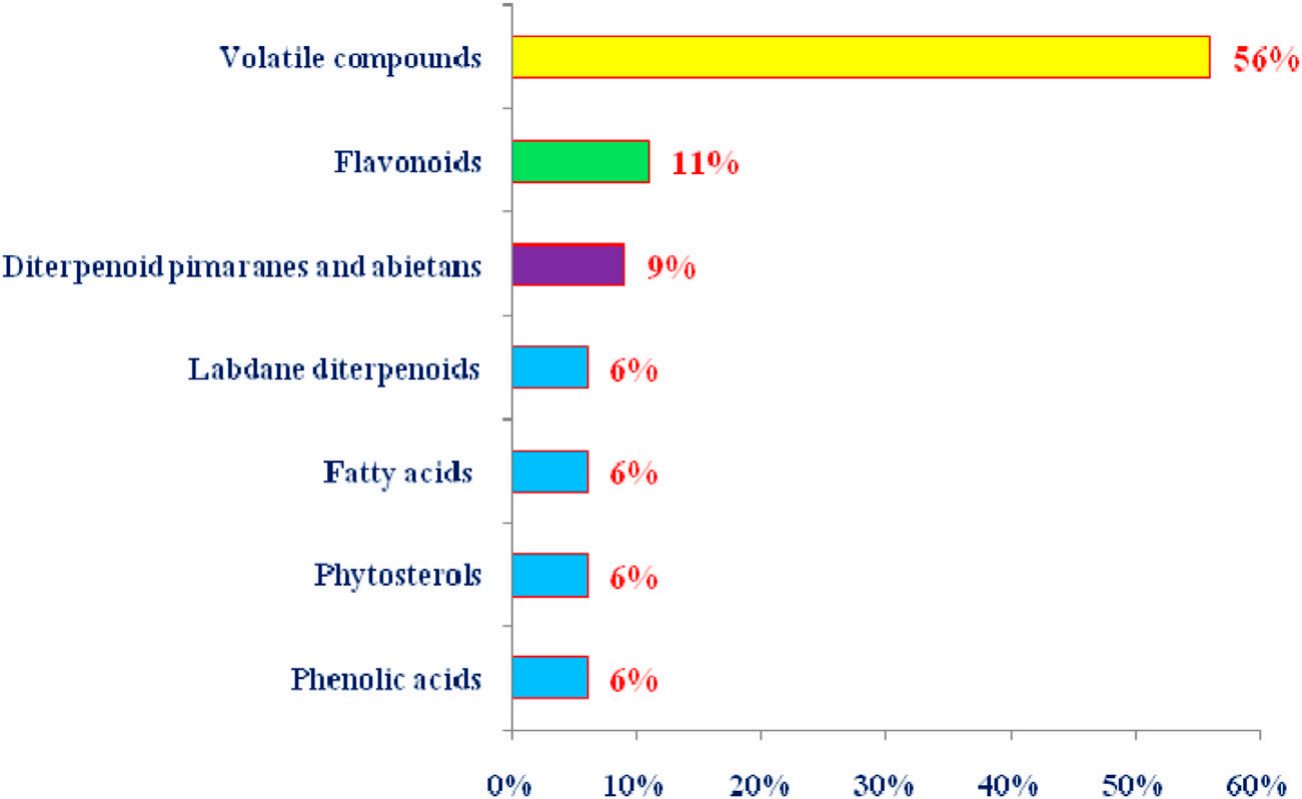
Summary of the phytoconstituents from *T. articulata* by class of natural products (The percentage of each class was calculated based on the total number of chemical components).

*T. articulata* has been proven to be a rich repository of essential oil that might obtained from various parts, including leaves, stems, cones, leafy twigs, using advanced techniques such as microwave-assisted hydrodistillation, supercritical fluid extraction, or conventional ones such as hydrodistillation using a Clevenger-type apparatus. The chemical composition of *T. articulata*’s essential oil extracted from woody branches, cones, resin, woods, wood sawdust, and roots has been the subject of numerous studies in North Africa countries and Malta (Buhagiar et al., 2000; Djouahri et al., 2016; Ben Ghnaya et al., 2016; Harmouzi et al., 2016). They reported that the chemical composition showed slight variations depending on the origin and organs from which they were originated, with bornyl acetate and camphor being the dominant encountered chemical components (Table 3). So far, the EOs contained mainly monoterpene hydrocarbons, oxygenated monoterpenes, sesquiterpene hydrocarbons, and oxygenated sesquiterpene (Figure 5) (Abi-Ayad et al., 2013). Bornyl acetate, camphor, and *α*-pinene are essential oil markers in leaf identification at various phenological stages (Bahri et al., 2015). In this sense, Sadiki et al. (2018) reported that the chemical composition of the essential oil from leaves of *T. articulata* collected in the eastern region of Morocco was dominated by monoterpene hydrocarbons (47.08%), with *α*-pinene (22.68%), bornyl acetate (16.87%), camphor (14.52%), and limonene (7.34%). These findings are in agreement with those obtained by Bahri et al. (2015), who examined the EO of *T. articulata* leaves (fresh and dried) collected in Algeria. They indicated that *α*-pinene (36.1%; 44.1%), bornyl acetate (18.3%; 3.1%), camphor (1.7%; 20.1%), and limonene (2.9%; 5.0%) were the most representative constituents. Similar results were also mentioned by Achak et al. (2009), Bourkhiss et al. (2007), and Sadiki et al. (2018). These outcomes slightly disagree with those of Ben Ghnaya et al. (2016), who showed that the major constituents of the EO of *T. articulata* leaves collected in Nabeul, Tunisia, were *α*-pinene (56.21%), followed by 1,8-cineole (9.91%), isobornyl acetate (7.46%), and *β*-myrcene (3.08%). On the other hand, a large body of phytochemical studies revealed that the chemotype of plant leaves differed from wood sawdust, root, resin, and cones. For instance, the oxygenated monoterpenes *α*-campholenal, *trans*-pinocarveol, verbenone, and *cis*-verbenol were identified as the preponderant components of the EO of *T. articulata* cones taken from the area of Ain-Defla, Algeria (Djouahri et al., 2013), whereas *trans*-pinocarveol, fenchyl acetate, *p*-cymene-8-ol, and *β*-phellandrene were the main constituents of the EO of *T. articulata* cones collected from the area of Zaghouan, Tunisia (Tékaya-Karoui and Mighri, 2007). Indeed, differences in chemical composition can be linked to several factors, including the time of harvest and drying techniques, which may result in the destruction of small molecules with low vaporization points. The variability in soil nutrient concentrations and their storage in leaves can also lead to plenty of metabolic processes and the synthesis of various bio-products and volatile components (Djouahri et al., 2013).

**TABLE 3:** Chemical composition of *T. articulata* EOs and extracts.

Compound names	Plant organs	Characterization methods	References
**Flavonoids**			
Quercetin (quercetin-3-*O*-rhamnoside)	Cones, Leaves	LC–DAD–ESI–MSn, UPLC-PDA-MS, GC–MS,HPLC	Djouahri et al. (2014); Zidane et al. (2014); Dane et al. (2016); Rached et al. (2018)
Rutin			
(+)-Catechin			
(−)-Epicatechin			
Isoquercetin (quercetin-3-*O*-glucoside)			
Myricetin-*O*-pentosyl- *O*-hexoside			
Myricetin-3-*O*- rutinoside			
Myricetin-3-*O*- glucoside			
Myricetin-3-*O*- rhamnoside			
Kaempferol-3-*O*- rhamnoside			
Kaempferol-deoxyhexose			
Cupressuflavone			
Amentoflavone			
**Phenolic acids**			
Caffeic acid	Cones, Leaves	LC–MS, GC–MS	Djouahri et al. (2014); El Jemli, (2019)
Vanillic acid			
Rosmarinic acid			
Chlorogenic acid			
Syringic acid			
Salicylic acid			
**Volatile compounds**			
*α*-Pinene	Leaves, cones, resin	GC–MS, RI	El Moussaouiti et al. (2010); Abi-Ayad et al. (2013); Djouahri et al. (2014); Djouahri et al. (2016); Harmouzi et al. (2016); Saber et al. (2021)
*α*-Terpinyl acetate			
*α*-Campholenal			
*α*-Cedrene			
Thymol mono			
β-Caryophyllene			
Caryophyllene oxide			
Isobornyl acetate	Leaves, resin		
Limonene			
*α*-Copaene			
Borneol	Leaves, bark		
Carvacrol			
*α*-Terpineol	Bark, cones		
*trans*-Pinocarveol			
Verbenone			
*cis*-Verbenol			
Myrtenol			
4-Terpineol	Resin, bark		
Totarol	Burl wood, resin		
Bornyl acetate	Resin, cones		
Myrtenal	Leaves, cones		
*α*-Muurolol	Leaves, Burl wood		
3-Tert-butyl-4-methoxyphenol	Burl wood		
Cedrenol			
Sclarene			
*β*-Himachalene			
Cedran-diol			
*trans*-Verbenol	Bark		
*γ*-Muurolen			
Thymylisobutyrate			
Nerylisobutyrate			
Carotol			
Longifolene			
Elemol			
Humulene epoxide II			
Atiserene			
Fenchol			
Cyclosativene			
Camphor	Leaves		
*α*-Cadinol			
1-*Epi*-Cubenol			
*δ*-Cadinene			
Sibirene			
*β*-Biotol			
*β*-Oplopenone			
Valencene			
Germacrene D			
Myrcene			
Campholenal			
Isoledene			
Cedrol			
Cubenol			
Caryophylladienol			
Bourbonelol			
*γ*-Cadinene			
Isoledene			
*α*-Phellandrene			
Tricyclene			
*α*-Terpinolene			
*p*-Cymene mono			
*p*-Xylene	Cones		
2,4-thujadiene			
Linalool			
α-campholenal			
Pinocarvone			
*β*-Selinene	Resin		
Viridiflorene			
Carvone			
*α*-Gurjunene			
1-*Epi*-cubenol			
**Fatty acids and fatty alcohols**			
Dodecanoic acid	Trunk bark	GC-EI-MS, GC/MS	Jlizi et al. (2021)
2-Nonanol			
1-Hexadecene			
*cis*-Arteannuic acid			
n-Hexadecane			
**Phytosterols**			
*β*-Sitosterol	Cones	CGC	Saber et al. (2021)
Compesterol			
Brassicasterol			
Stigmasterol			
Cholesterol			
Δ-5-Avenasterol			
Δ-7-Avenasterol			
Δ-7-Stigmasterol			
**Diterpenoid pimaranes and abietans**			
Sandaracopimaric acid	Sandarac Resin	GC-MS	Sugimoto et al. (2006); Romero-Noguera et al. (2014)
Methylsandaracopimarate			
Methyl pimarate			
Sandaracopimarinol			
4-*Epi*-dehydroabietic acid			
Pimaric acid			
*trans*-Ferruginol			
*trans*-Ferruginylacetate			
Isopimaric acid			
Pimara-7,15-dien-3-one			
Laevopimaric acid			
**Labdane diterpenoids**			
*cis*-Communic acid	Sandarac Resin	GC-MS	Romero-Noguera et al. (2014)
*trans*-Communic acid			
Manool			
Totarol			
Agathalic acid			
Acetoxy agatholic acid			
Agathic acid			
Agatholic acid			
**Organic Composition**			
Lipids content (6.66%)	Air-dried leaf	Kjeldahl method	Achak et al. (2009)
Total nitrogen (0.07%)			
Protein (0.44%)			
Wax and Greases (6%)			

### Phenolic compounds

There are relatively few studies that have been devoted to investigating the plant’s phenolic compounds. These studies identified 21 phenolic compounds from leaf and cones crude extracts (Djouahri et al., 2014; Zidane et al., 2014; Dane et al., 2016; Rached et al., 2018). These compounds are represented by phenolic acids (gallic and caffeic acids), flavonoid aglycones, and flavonoid glycosides and they were identified from 100% MeOH, 100% EtOH, and 70% EtOH extracts of air-dried cones of *T. articulata* (Figure 5; Table 3) (Djouahri et al., 2014). On the other hand, the aqueous extract of *T. articulata* air-dried cones was shown to contain exclusively tannic and gallic acids, while caffeic acid was lacking (Djouahri et al., 2014). Moreover, nine phenolic compounds have been identified in crude aqueous extract, ethyl acetate fraction, and butanol fraction of *T. articulata* leaves, including three flavan-3-ols and six flavanols (Rached et al., 2018). Accordingly, flavan-3-ols were the most common phenolic chemicals in the three samples, constituting up to 71% of the total phenolic content. B-type *epi*catechin dimer was the primary constituent in the aqueous crude extract and the butanol fraction, whereas catechin was by far the most abundant component in the ethyl acetate fraction (Rached et al., 2018). Flavonols made up an average of 29% of the total phenolic content, with the ethyl acetate fraction having the highest composition and the glycoside myricetin-3-*O*-rhamnoside as the major constituent (Rached et al., 2018). Furthermore, cupressuflavone and amentoflavone, two naturally occurring biflavonoids with hydroxyl substituent, were identified in the methanol extract and ethyl acetate extract (decoction) of *T. articulata* leaves, whereas they were absent in the aqueous extract (Zidane et al., 2014).

### Diterpenoid pimaranes, labdanes and abietans of *T. articulata* sandarac resin

*T. articulata* resin is a highly balsamic and viscous substance with a light yellow color and a melting point of about 145°C (Kononenko, 2017). It is secreted from specialized internal and external structures and released for different purposes including, defense, protection against predators, and interaction with its surrounding environment (Zona, 2004; Kononenko, 2017). The use of sandarac resin is traced back to 1800 BC, and since then, it has been frequently used for artistic, therapeutic, and ritual purposes (Romero-Noguera et al., 2014; Kononenko, 2017). Previous research on the chemical composition of sandarac resin revealed that communic acid, a natural bicyclic diterpenoid with a labdane skeleton makes up over 70% of the resin. The communic acid was shown to be responsible for the polar properties and poor solubility of the resin after aging (Romero-Noguera et al., 2014). So far, a total of 42 compounds were identified during the chemical analysis of sandarac resin (Sugimoto et al., 2006; Romero-Noguera et al., 2014; Jlizi et al., 2018). These compounds consist primarily of labdane diterpenes such as *cis*-communic acid, *tran*s-communic acid, and agathalic acid. Sandarac resin also contains six pimaranes and abietans diterpenoids, including sandaracopimarinol, sandaracopimaric acid, and laevopimaric acid, as well as small amounts of phenolic components such as totarol and manool (Figure 5; Table 3) (Sugimoto et al., 2006; Romero-Noguera et al., 2014).

## *In silico* toxicity prediction of phenolic compounds as well as the major volatile compounds found in *T. articulata*

The study of the toxicity of a candidate compound is a crucial step in the drug development process before proceeding into clinical trials. Thus, the in silico toxicity tools emerged as a time-saving and inexpensive alternative to animal experiments (Bai and Abernethy, 2013). On the other hand, given the scarcity of toxicological research on the plant species, we have relied on the online web Tool ProTox-II (http://tox.charite.de/protox_II, accessed on 26 April 2022) to predict the organ toxicity (hepatotoxicity) (since the liver is the organ where these compounds are metabolized) as well as four toxicological endpoints, namely cytotoxicity, mutagenicity, carcinogenicity, and immunotoxicity of the phenolic compounds alongside the major volatile compounds found in different *T. articulata* extracts. The predicted descriptors are listed in Table 4.

**TABLE 4:** The predicted organ toxicity and toxicological endpoints of phenolic compounds and the main volatile compounds using the ProTox-II server.

Compounds	Molecular weight	Predicted LD_50_ (mg/kg)	Toxicity class	Organ toxicity (% probability)		Toxicity Endpoints (% probability)		
				Hepatotoxicity	Carcinogenicity	Immunotoxicity	Mutagenicity	Cytotoxicity
**Phenolic acids**								
Gallic acid	170.12	2000	IV	Inactive (61)	Active (56)	Inactive (99)	Inactive (94)	Inactive (91)
Caffeic acid	180.16	2,980	V	Inactive (57)	Active (78)	Inactive (50)	Inactive (98)	Inactive (86)
Vanillic acid	168.15	2000	IV	Inactive (55)	Inactive (64)	Inactive (97)	Inactive (96)	Inactive (93)
Rosmarinic acid	360.32	5,000	V	Inactive (62)	Inactive (66)	Active (93)	Inactive (85)	Inactive (90)
Chlorogenic acid	354.31	5,000	V	Inactive (72)	Inactive (68)	Active (99)	Inactive (93)	Inactive (80)
Syringic acid	198.17	1700	IV	Inactive (58)	Inactive (70)	Inactive (97)	Inactive (93)	Inactive (97)
Salicylic acid	138.12	1,034	IV	Active (51)	Inactive (67)	Inactive (99)	Inactive (98)	Inactive (86)
**Flavonoids**								
Quercetin	302.24	159	III	Inactive (69)	Active (68)	Inactive (87)	Active (51)	Inactive (99)
Isoquercetin	464.38	5,000	V	Inactive (82)	Inactive (85)	Active (66)	Inactive (76)	Inactive (69)
Rutin	610.52	5,000	V	Inactive (80)	Inactive (91)	Active (98)	Inactive (88)	Inactive (64)
(+)-Catechin	290.27	10,000	VI	Inactive (72)	Inactive (51)	Inactive (96)	Inactive (55)	Inactive (84)
(−)-Epicatechin	290.27	10,000	VI	Inactive (72)	Inactive (51)	Inactive (96)	Inactive (55)	Inactive (84)
Cupressuflavone	538.46	3,919	V	Inactive (76)	Inactive (69)	Inactive (92)	Inactive (71)	Inactive (98)
Amentoflavone	538.46	3,919	V	Inactive (76)	Inactive (69)	Active (51)	Inactive (71)	Inactive (98)
Myricetin-3-*O*- rutinoside	626.52	5,000	V	Inactive (80)	Inactive (91)	Active (99)	Inactive (88)	Inactive (64)
Myricetin-3-*O*- glucoside	470.3	5,000	V	Inactive (82)	Inactive (85)	Active (79)	Inactive (76)	Inactive (79)
Kaempferol-3-*O*- rhamnoside	432.38	5,000	V	Inactive (73)	Active (50)	Active (92)	Inactive (71)	Inactive (93)
**Main volatile compounds**								
Bornyl acetate	196.29	3,100	V	Inactive (58)	Inactive (62)	Inactive (94)	Inactive (94)	Inactive (67)
Camphor	152.23	775	IV	Inactive (72)	Inactive (68)	Inactive (96)	Inactive (94)	Inactive (61)
*α*-Pinene	136.23	3,700	V	Inactive (86)	Inactive (60)	Inactive (99)	Inactive (93)	Inactive (75)

Class I: fatal if consumed (LD50 ≤ 5); Class II: fatal if consumed (5 < LD50 ≤ 50); Class III: toxic if consumed (50 < LD50 ≤ 300); Class IV: harmful if consumed (300 < LD50 ≤ 2000); Class V: may be harmful if consumed (2000 < LD50 ≤ 5,000); Class VI: non-toxic (LD50 > 5,000).

Regarding organ toxicity, results revealed that salicylic acid was assumed to be hepatotoxic, while the other compounds were expected to be hepatotoxic-free. Proceeding *in vivo* and *in vitro* studies have corroborated the previous *in silico* findings showing that salicylic acid may occasionally induce lipid peroxidation and severe liver damage, especially under oxidative stress (Doi and Horie, 2010). When it came to toxicological endpoints, all the chemical constituents were predicted to be devoid of any cytotoxicity. Quercetin, a naturally occurring flavonol, showed two toxicological endpoints, namely mutagenicity and carcinogenicity, while gallic acid, caffeic acid, and kaempferol-3-*O*-rhamnoside were predicted as both immunotoxic and carcinogenic. Previous *in vivo* and *in vitro* investigations revealed that quercetin displayed positive mutagenicity and carcinogenicity, which is consistent with in silico prediction (Ukoroije and Otayor, et al., 2020 Utesch et al., 2008). The median lethal dose (LD_50_) values were estimated to be between 159 and 5,000 mg/kg (Table 4). According to the globally harmonized system of classification of labeling of chemicals (GHS) (Drwal et al., 2014), the majority of phenolic acids fall in the category of ‘harmful if consumed’ (Toxicity class IV), while caffeic acid, rosmarinic acid, and chlorogenic acid were categorized as ‘may be harmful if swallowed’ (Toxicity class V) with LD_50_ values of 2,980, 5,000, and 5,000 mg/kg, respectively. Similarly, most flavonoids were assigned to toxicity classes IV or V, except for quercetin, which was allocated in category III (Toxic if swallowed). However, in depth studies are needed to evaluate the toxicity of *T. articulata* due to the synergetic effects and possible interactions its multiple components with several proteins and body organs.

## Biological activities of *T. articulata*

### Cytotoxicity properties

**FIGURE 6:**
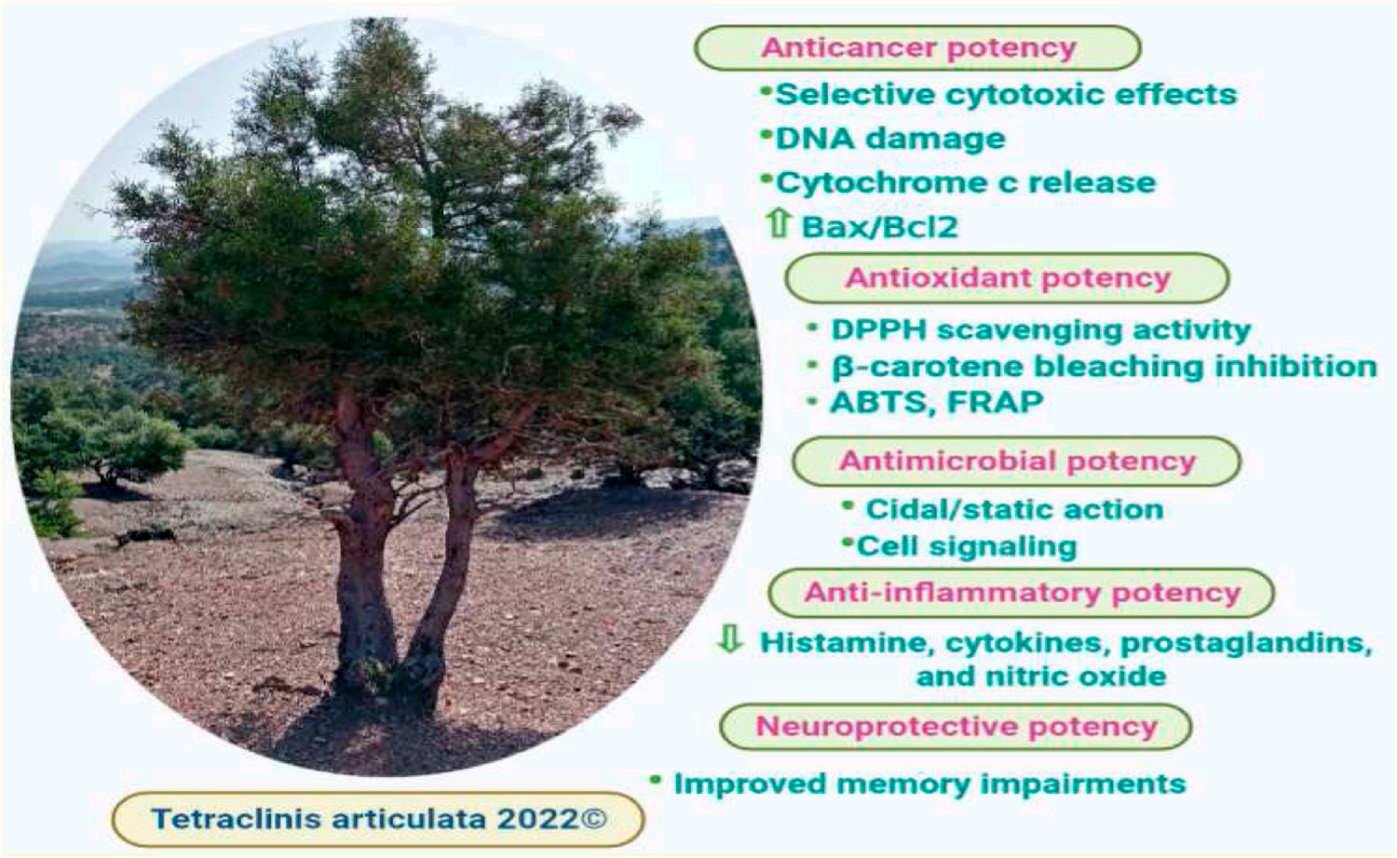
Summary of biological activities of *T. articulata.*

The *in vitro* anticancer activity of *T. articulata* extracts was examined against various cancer cell lines, offering data on the bioactivity of both the extract and the individual compounds (Figure 6). To overcome multidrug resistance in MDA-MB-231 breast cancer cells and investigate whether *T. articulata* essential oil possesses substantial *in vitro* cytotoxic properties (Table 5). Jlizi et al. (2021) tested the *in vitro* cytotoxic activity of *T. articulata* trunk bark essential oil against MDA-MB-231 breast cancer and SW620 colon cancer using the cell viability assay (MTT). They reported that the EO exhibited moderate dose-dependent cytotoxic potency against both cell lines. They also recorded IC_50_ values of 25.7 and 83.0 μg/ml against SW620 and MDA-MB-231cells, respectively, after 48 h of exposure. To improve the extract’s cytotoxicity, the authors fractionated the EO on a silica gel column using a step gradient of hexane/ethyl acetate and reassessed each fraction against both cell types. Accordingly, the fraction hexane/ethyl acetate (90:10) exhibited better activity against SW620 cells, while the fraction (80:20) was effective against MDA cells. They correlated the cytotoxic effect with the presence of particular sesquiterpene compounds, such as caryophyllene and caryophyllene oxide. Using the same method, Rached et al. (2018) reported that the crude aqueous extract of *T. articulata* and its fractions disclosed good cytotoxic effects toward four human cancer cells, namely CI-H460 (non-small cell lung cancer), HeLa (cervical carcinoma), MCF-7 (breast carcinoma), and HepG2 (hepatocellular carcinoma), with ethyl acetate fraction being the most prominent against all the tested cell lines. They reported that the ethyl acetate fraction was the richest in total flavonoids 93.1 mg/g, compared to 21.2 mg/g in the crude aqueous extract, and ascribed the effects to certain flavonoids, such as flavonols (catechin, and *epi*catechin). To seek cytotoxic agents with high selectivity towards cancer cells to counteract metastatic cancers, Calderón-Montaño et al. (2021) screened 65 extracts prepared from 45 plants growing in Spain for their cytotoxic capacity against normal lung cells (MRC-5) and lung cancer cells (A549). They reported that *T. articulata* leaves ethanol/ethyl acetate/water (1:1:1) extract exhibited the highest activity against A549 cells (IC_50_ = 0.37 ± 0.03 μg/ml); Meanwhile, displaying less toxicity towards lung normal cells IC_50_ = 129.5 ± 64.0 μg/ml, (high selectivity). Moreover, the diterpene *trans*-communic acid and *cis*-trans mixture were isolated from the resin of *T. articulata* and screened for their cytotoxic potential against HeLa and A549 cell lines, following the MTT assay. Both *trans*-communic acid and *cis*-trans mixture exhibited potent cytotoxic effects on the HeLa cell line, with IC_50_ values of 12.8 ± 0.5 and 09.5 ± 1.0, respectively (Jlizi et al., 2018). Moreover, the essential oil of this tree contained thymoquinone, a monoterpene with promising anti-cancer properties; It has been reported to induce apoptosis in many solid and liquid tumors through increasing Bax/Bcl2 ratio and the expression of pro-apoptotic molecules, including caspase-3 and caspase-9, triggering cytochrome c release, DNA damage, and through P53 dependent pathway (Ahmad et al., 2019). These studies disclosed the anticancer potential of *T. articulata*; however, in-depth *in vivo*, toxicological, and clinical trials are mandatory to ensure its efficacy and safety.

**TABLE 5:** Summary of cytotoxic, neuroprotective, anti-inflammatory, and antioxidant potencies of various *T. articulata* extracts.

Extract	Used method	Country	Key results	References
**Leaves**				
Cytotoxic activities				
Ethanol/ethyl acetate/water extract	A64-CLS, AN3Ca, Calu-1, GAMG, HNO97, HT29, KATO II, MDA-MB-231, MeWo, PC-3, Sk-Br-3, Sk-OV-3, T24, UACC-62, A549 cell lines	Spain	IC_50_ ranged between 0.37 ± 0.03 and 4.9 μg/ml; the extract exhibited high selectivity and cytotoxic effect against lung cancer cells A549	Calderón-Montaño et al. (2021)
Crude aqueous extract	MCF-7, NCI-H460, HeLa, HepG2 cell lines	Algeria	IC_50_ ranged between 98 ± 8 and 307 ± 27 μg/ml	Rached et al. (2018)
Ethyl acetate fraction			IC_50_ ranged between 59 ± 3 and 189 ± 7 μg/ml	
Butanol fraction			IC_50_ ranged between 76 ± 4 and 209 ± 15 μg/ml	
**Anti-Alzheimer activity**				
Essential oil	Y-maze task, Radial arm maze task	Morocco	The EO vapor at concentrations of 1 and 3% reversed the reduction in Aβ1-42-induced spontaneous alternation, while in the radial arm maze; the inhalation of the EO significantly rectified the working and References memory errors	Sadiki et al. (2018)
**Antioxidant activities**				
Aqueous extract	DPPH scavenging activity	Algeria	IC_50_ = 12.7 ± 0.7 μg/ml	Rached et al. (2018)
	I		IC_50_ = 12.9 ± 0.1 μg/ml	
	Reducing power			
	β-carotene bleaching inhibition		IC_50_ = 1,180 ± 19 μg/ml	
	TBARS inhibition		IC_50_ = 4.8 ± 0.4 μg/ml	
Aqueous extract	DPPH scavenging activity	Morocco	IC_50_ = 27.38 ± 0.02 μg/ml	El Jemli et al. (2016)
	II.		IC_50_ = 32.92 ± 0.56 μg/ml	
	ABTS			
	FRAP		IC_50_ = 47.12 ± 0.15 μg/ml	
Essential oil (Hydrodistillation)	DPPH scavenging activity	Algeria	IC_50_ = 517.65 ± 1.21 μg/ml	Djouahri et al. (2013)
	III.		IC_50_ = 45.12 ± 0.14 μg/ml	
	Reducing power			
Essential oil (Microwave-assisted hydrodistillation)	DPPH scavenging activity		IC_50_ = 191.72 ± 1.22 μg/ml	
	IV.		IC_50_ = 30.19 ± 0.15 μg/ml	
	Reducing power			
**Anti-inflammatory**				
Aqueous extract	Nitric oxide (NO) production	Algeria	IC_50_ = 241 ± 4 μg/ml	Rached et al. (2018)
Essential oil (Hydrodistillation)	Lipoxygenase inhibition assay	Algeria	IC_50_ = 72.55 ± 0.14 μg/ml	Djouahri et al. (2013)
	V		IC_50_ = 61.56 ± 0.19 μg/ml	
	Xanthine oxidase (XO) inhibition assay			
Essential oil (Microwave-assisted hydrodistillation)	Lipoxygenase inhibition assay		IC_50_ = 79.88 ± 0.13 μg/ml	
	VI.		IC_50_ = 74.95 ± 0.15 μg/ml	
	Xanthine oxidase (XO) inhibition assay			
**Trunk bark**				
Essential oil (Cytotoxic activity)	MDA-MB-23, SW620 cell lines	Tunisia	The EO exhibited a cytotoxic effect against both cell lines MDA-MB-23, SW620, with IC50 values of 83.0 and 25.7 μg/ml, respectively	Jlizi et al. (2021)

### Antioxidant activity

Recently, there has been a keen interest in identifying new plant-based antioxidants to protect the human body from free radical-related disorders such as diabetes, cancer, cardiovascular and neurodegenerative diseases, atherosclerosis, arthritis, asthma, anemia, inflammation, and so forth (Pham-Huy et al., 2008; Yassir et al., 2022). Many research studies showed that various plant species contained significant amounts of polyphenolic compounds and flavonoids, which could also be applied as promising antioxidant agents in dietary supplements and as food additives to postpone or prevent rancidity. Therefore, the antioxidant properties of *T. articulata* have been the subject of a great deal of research in North Africa countries. However, they all reported low-to-moderate antiradical activity of the essential oil. Meanwhile, they noticed a potent antioxidant capacity of the aqueous and acetone extracts, as mentioned in Table 5. Djouahri et al. (2016) evaluated the antioxidant potency of leaves essential oil of *T. articulata* from Algeria using scavenging reducing power activities and DPPH free radicals. According to the authors, the extract exhibited poor antiradical activity with an IC_50_ value of 517.6 ± 1.75 μg/ml, which was very weak compared with both standards BHA and BHT (IC_50_ = 21.28 ± 0.12 μg/ml and 12.76 ± 0.08 μg/ml, respectively). Another investigation that corroborated the previous findings was carried out by Rabib et al. (2020). They reported that the essential oil of *T. articulata* leaves from Morocco has moderate antioxidant power compared with conventional antioxidants at a dose of 2 mg/ml. It is widely known that the antiradical activity of a plant extract is strongly correlated with the total amounts of polyphenolic compounds and flavonoids, which are highly stable molecules capable of scavenging free radicals by H-atom donating. However, essential oils, particularly those obtained by hydrodistillation, are utterly bereft of phenolic compounds, which may explain why *T. articulata* essential oil exhibited poor antioxidant efficacy (Sliti et al., 2016). On the other hand, El Jemli et al. (2016) tested the *in vitro* antioxidant power of leaf aqueous extract of *T. articulata* using DPPH, ABTS, and FRAP. The authors reported that the extract showed strong scavenging activity displaying IC_50_ values of 27.38 ± 0.02 μg/ml (DPPH), 32.92 ± 0.56 μg/ml (ABTS) and 47.12 ± 0.15 μg/ml (FRAP). They also indicated a strong link between the antioxidant capacity and phenolic content of the extract (11.78 ± 0.30 µg (QE)/mg edw), demonstrating that phenolic compounds are major contributors to antioxidant properties of *T. articulata* aqueous extract. The previous findings were backed up by the investigation conducted by Ben Jemia et al. (2012). The authors used four different assay systems, namely DPPH, reducing power, β-carotene/linoleic acid, and metal chelating activity test to assess the *in vitro* antioxidant activities of *T. articulata* leaves 80% aqueous acetone extract. They indicated that the extract exhibited potent free radical scavenging activity in the DPPH system displaying an IC_50_ value of 5.5 mg/ml, which was two times higher than the standard positive control (BHT). They associated the powerful antioxidant effect with the high amount of the total phenolics, flavonoids, and condensed tannins in the extract. The previous findings supported the possible application of *T. articulata* extracts in the fields of food industry and cosmetics as well as to manage several free radical-related diseases. However, further *in vivo* studies and clinical trials are mandatory to validate the species safety and define appropriate doses.

### Anti-inflammatory activity

To lend credence to the ethnomedicinal uses of *T. articulata* as an anti-inflammatory agent, El Jemli et al. (2016) tested the *in vivo* anti-inflammatory activity of *T. articulata* leaves essential oils on carrageenan and trauma-induced rats paw edema. They reported that the EO exhibited dose-dependent anti-inflammatory effects toward carrageenan-induced rat paw edema with a maximal effect (68.42% inhibition) achieved at 200 mg/kg after 3h, which was almost identical to the standard anti-inflammatory agent indomethacin (72.63% inhibition). They also mentioned dose-dependent anti-inflammatory effects on trauma-induced rat paw edema displaying substantial inhibition (84.51%) at the concentration of 200 mg/kg, which was also the same as indomethacin (84.08% inhibition). They hypothesized that the anti-inflammatory effects could be associated with the synthesis inhibition or release of cyclooxygenase products, such as histamine, cytokines, prostaglandins, and nitric oxide. They also indicated that the inflammatory mediators’ inhibition is likely produced by specific components of the essential oils like monoterpene hydrocarbons and oxygenated monoterpenes. Furthermore, *T. articulata* leaves have been widely used in North Africa’s folk medicine system to cure inflammatory infections. To validate the ethnomedicinal uses, Rached et al. (2018) evaluated the *in vitro* anti-inflammatory potential of *T. articulata* leaves aqueous extract on LPS-induced NO production by Murine macrophage. They found that the extract and its organic fractions ethyl acetate fraction and butanol fraction considerably reduced NO production. They linked the significant anti-inflammatory effect with the richness of the plant extract with flavonoids, especially catechin, *epi*catechin, and myricetin-3-*O*-rhamnoside.

### Antiurolithiatic activity

The antiurolithiatic activity of *T. articulata* aqueous extract (infusion), collected in Algeria, was tested *in vitro* on the formation and inhibition of calcium oxalate monohydrate using polarized light microscopy. According to the results, the extract prevented urinary stones growth, with the highest inhibitory effects (87.94 and 84.12%) noticed at the concentration of 100 and 50%, respectively. However, in-depth *in vivo* and *in vitro* studies are needed to elucidate the curative and prophylactic potential of *T. articulata* extracts in preventing urolithiasis (Beghalia et al., 2007).

### Neuroprotective activity

There are relatively few studies on the neuroprotective effect of *T. articulata* to back up the traditional applications and provide scientific evidence for further studies. Sadiki et al. (2018) investigated the potency of *T. articulata* leaves essential oil in amyloid-*β* peptide 1-42 (A*β*1-42)-induced spontaneous alternation, working, and reference memory errors in rats, using the Y-maze and radial arm maze tests. Results indicated that in the Y-maze test, the EO vapor at concentrations of 1 and 3% reversed the reduction in A*β*1-42-induced spontaneous alternation, which was almost similar to the donepezil effect (Sadiki et al., 2018). Results also showed that in the radial arm maze, the inhalation of the EO significantly rectified the working and reference memory errors. Moreover, the EO attenuated A1-42-induced cholinergic deficits and lowered acetylcholinesterase activity in the rat hippocampus. Similarly, Postu et al. (2022) assessed the *in vivo* neuroprotective properties of *T. articulata* EO (3%) in the rat model of amyloid-beta 1-42 (A1-42)-induced Alzheimer’s disease (AD) after administering the EO by inhalation once daily for 21 days. Results showed that the EO substantially attenuated memory impairments, mainly through improving the expressions of Brain-derived neurotrophic factor (BDNF) and down-regulating the expression of interleukin-1*β* (IL-1β) gene expressions associated with A*β*1-42-induced toxicity in the rat model. These findings proved that the plant EO might be a natural neuroprotective agent against A*β*1-42-induced neurotoxicity.

### Antimicrobial activity

*T. articulata* has been widely used in folk medicine as a natural antibiotic agent to manage intestinal and urinary tract infections (Belgacem et al., 2021). These empirical practices were the springboard to seek better alternatives to standard antimicrobial drugs, especially with the harmful effects attributed to these antibiotics and the growth of multidrug resistance in a full range of Gram-positive and Gram-negative bacteria. Previous studies have described the antimicrobial activity of crude extracts from *T. articulata*. The antibacterial activity of the plant extracts was evaluated using disc diffusion and the disc volatilization assays, agar well diffusion, and broth microdilution procedures. As indicated in Table 6, microbial growth inhibition zones and percentages, as well as minimum inhibitory concentrations (MICs), showed that *T. articulata* exhibited a potent bacteriostatic effect.

**TABLE 6:** Summary of the antimicrobial potency of *T. articulata* extracts.

Extract	Tested strains	Key results	Country	References
Sawdust of the root burl				
**EO**	09 Strains of *Staphylococcus aureus*	MIC ranging between 0.19 and 48 nL/ml and IZ ranging between 25 and 44 mm (*S. aureus* were highly susceptible by the essential oil)	Morocco	Achmit et al. (2021)
	*Enterococcus faecalis*	MIC = 5 μL/ml; Φ mm = 19		Talbaoui et al. (2016)
	*Escherichia coli*	MIC = 10 μL/ml; Φ mm = 10		
	*Klebsiella pneumoniae*	MIC = 10 μL/ml; Φ mm = 17		
	*Streptococcus* D	MIC = 10 μL/ml; Φ mm = 13		
	*Escherichia coli*	MIC = 1.25 μL/ml; MBC = 1.25 µL/ml; Φ mm = 17		El Moussaouiti et al. (2010)
	*Streptococcus* D	MIC = 1.25 μL/ml; MBC = 1.25 µL/ml; Φ mm = 13		
	*Enterococcus faecalis*	MIC = 1.25 μL/ml; MBC = 1.25 µL/ml; Φ mm = 12		
	*Klebsiella pneumoniae*	MIC = 1.25 μL/ml; MBC = 20 µL/ml; Φ mm = 13		
**Branches**				
**EO**	*Escherichia coli*	MIC = 2.5 μL/ml; Φ mm = 19.16 ± 0.57	Morocco	Sadiki et al. (2018)
	*Klebsiella pneumoniae*	MIC = 80 μL/ml; Φ mm = 6.66 ± 0.76		
	*Acinetobacter baumannii*	MIC = 2.5 μL/ml; Φ mm = 17 ± 0.28		
	*Staphylococcus aureus*	MIC = 40 μL/ml; Φ mm = 9.26 ± 0.30		
**Leaves**				
**EO**	*Staphylococcus aureus* (ATCC 25923)	MIC = 7.5 μL/ml; Φ mm = 22	Algeria	Abi-Ayada et al. (2011)
	*Bacillus cereus* (ATCC11778)	MIC = 5 μL/ml; Φ mm = 60.5		
	*Pseudomonas aeruginosa* (ATCC27853)	Φ mm = 1		
	*Escherichia coli* (ATCC 25922)	MIC = 10 μL/ml; Φ mm = 19		
	*Escherichia coli* (ATCC25921)	Φ mm = 1		
	*Staphylococcus aureus* (ATCC 29213)	Φ mm = 48 ± 00; MIC and MBC = 1.56 ± 0.00 μg/ml	Morocco	Rabib et al. (2020)
	*Escherichia coli* (ATCC 25922)	Φ mm = 8 ± 00; MIC and MBC = 25 ± 0.00 μg/ml		
	*Pseudomonas aeruginosa* (ATCC 27853)	Φ mm = 30 ± 00; MIC and MBC = 6.25 ± 0.00 μg/ml		
	*Staphylococcus aureus*	Φ mm = 35 ± 00; MIC and MBC = 6.25 ± 0.00 μg/ml	Morocco	Rabib et al. (2019)
	*Escherichia coli*	Φ mm = 7 ± 00; MIC and MBC = 50 ± 0.00 μg/ml		
	*Pseudomonas aeruginosa*	Φ mm = 16 ± 00; MIC and MBC = 6.25 ± 0.00 μg/ml		
	*Escherichia coli* (ATCC 25922)	Φ mm = 7.0 ± 0.1; MIC = 1.0 μg/ml	Algeria	Chikhoune et al. (2013)
	*Pseudomonas aeruginosa* (ATCC 27853)	Φ mm = 7.5 ± 0.2; MIC = 0.4 μg/ml		
	*Staphylococcus aureus* (ATCC 25923)	Φ mm = 22.0 ± 0.9; MIC = 0.2 μg/ml		
	*Botrytis cinerea*	The EO demonstrated significant mycelial growth inhibition, with *Botrytis cinerea* being the most susceptible to the EO (71.17%) compared to the control	Tunisia	Ben Ghnaya et al. (2016)
	*Fusarium avenaceum*			
	*Fusarium oxysporum*			
	*Fusarium culmorum*			
	*Fusarium solani*			
**Cones**				
**EO**	*Escherichia coli* (ATCC 25922)	Φ mm = 10.0 ± 0.2; MIC = 0.4 μg/ml	Algeria	Chikhoune et al. (2013)
	*Pseudomonas aeruginosa* (ATCC 27853)	Φ mm = 12.0 ± 0.2; MIC = 0.2 μg/ml		
	*Staphylococcus aureus* (ATCC 25923)	Φ mm = 17.0 ± 0.6; MIC = 0.4 μg/ml		
**Aqueous**	*Listeria monocytogenes* (CIP82110)	Φ mm = 40.00 ± 0.57; MIC = 0.25 ± 0.00 μg/ml		
	*Staphylococcus aureus* (CIP 7625)	Φ mm = 14.00 ± 0.50; MIC = 15 ± 0.00 μg/ml		
	*Salmonella enterica* (E32)	Φ mm = 13.00 ± 0.50; MIC = 20 ± 0.00 μg/ml		
	*Escherichia coli* (ATTC 10536)	Φ mm = 12.50 ± 0.29; MIC = 30 ± 0.00 μg/ml		
	*Klebsiella pneumoniae* (CIP 8291)	Φ mm = 15.00 ± 0.29; MIC = 10 ± 0.00 μg/ml		
	*Pseudomonas aeruginosa* (CIPA 22)	Φ mm = 14.50 ± 0.29; MIC = 10 ± 0.00 μg/ml		
**MeOH 100%**	*Listeria monocytogenes* (CIP 82110)	Φ mm = 55.00 ± 1.00; MIC = 0.1 ± 0.0 μg/ml ^¥^		
	*Staphylococcus aureus* (CIP 7625)	Φ mm = 12.50 ± 0.5; MIC = 30 ± 0.00 μg/ml		
	*Salmonella enterica* (E32)	Φ mm = 12.17 ± 0.50; MIC = 30 ± 0.00 μg/ml ^¥^		
	*Escherichia coli* (ATTC 10536)	Φ mm = 12.00 ± 0.16; MIC = 30 ± 0.00 μg/ml ^¥^		
	*Klebsiella pneumoniae* (CIP 8291)	Φ mm = -		
	*Pseudomonas aeruginosa* (CIPA 22)	Φ mm = 14.50 ± 0.50; MIC = 10 ± 0.00 μg/ml		
**EtOH 70%**	*Listeria monocytogenes* (CIP 82110)	Φ mm = 42.00 ± 0.66; MIC = 0.25 ± 0.00 μg/ml ^γ^	Algeria	Djouahri et al. (2014)
	*Staphylococcus aureus* (CIP 7625)	Φ mm = 29.50 ± 0.50; MIC = 0.5 ± 0.00 μg/ml ^γ^		
	*Salmonella enterica* (E32)	Φ mm = 15.00 ± 0.50; MIC = 10 ± 0.00 μg/ml ^γ^		
	*Escherichiacoli* (ATTC 10536)	Φ mm = 14.50 ± 0.50; MIC = 10 ± 0.00 μg/ml ^γ^		
	*Klebsiella pneumoniae* (CIP 8291)	Φ mm = 20.00 ± 0.29; MIC = 1 ± 0.00 μg/ml ^γ^		
	*Pseudomonas aeruginosa* (CIPA 22)	Φ mm = 16.50 ± 0.29; MIC = 5 ± 0.00 μg/ml ^γ^		
**EtOH 100%**	*Listeria monocytogenes* (CIP 82110)	Φ mm = 28.00 ± 0.50; MIC = 0.5 ± 0.00 μg/ML ^δ^		
	*Staphylococcus aureus* (CIP 7625)	Φ mm = 11.50 ± 0.29; MIC = 40 ± 0.00µg/ml ^δ^		
	*Salmonella enterica* (E32)	Φ mm = 10.00 ± 0.50; MIC = 50 ± 0.00 µg/ml ^δ^		
	*Escherichia coli* (ATTC 10536)	Φ mm = 10.00 ± 0.50; MIC = 50 ± 0.00µg/ml ^δ^		
	*Klebsiella pneumoniae* (CIP 8291)	Φ mm = 12.00 ± 0.50; MIC = 30 ± 0.00µg/ml ^δ^		
	*Pseudomonas aeruginosa* (CIPA 22)	Φ mm = 11.50 ± 0.50; MIC = 40 ± 0.00µg/ml ^δ^		
**EO**	*Listeria monocytogenes* (CIP 82110)	Φ mm = 14.50 ± 0.50; MIC = 10 ± 0.00 µg/ml		
	*Staphylococcus aureus* (CIP 7625)	Φ mm = 17.50 ± 0.50; MIC = 5 ± 0.00 µg/ml		
	*Salmonella enterica* (E32)	Φ mm = 13.50 ± 0.50; MIC = 20 ± 0.00 µg/ml		
	*Escherichiacoli* (ATTC 10536)	Φ mm = 11.50 ± 0.16; MIC = 40 ± 0.00 µg/ml		
	*Klebsiella pneumoniae* (CIP 8291)	Φ mm = 10.50 ± 0.29; MIC = 60 ± 0.00 µg/ml		
	*Pseudomonas aeruginosa* (CIPA 22)	Φ mm = 12.50 ± 0.50; MIC = 30 ± 0.00 µg/ml		
**Fresh leaves**				
**EO**	*Escherichia coli*	Φ mm = 25; MIC = 268 µg/ml	Algeria	Bahri et al. (2015)
	*Klebsiella pneumoniae*	Φ mm = 23; MIC = 268 µg/ml		
	*Proteus mirabilis*	Φ mm = 25; MIC = 268 µg/ml		
	*Pseudomonas aeruginosa*	Φ mm = 25; MIC = 268 µg/ml		
	*Staphylococcus aureus*	Φ mm = 21; MIC = 516 µg/ml		
	*Candida albicans*	Φ mm = 23; MIC = 268 µg/ml		
**Dried leaves**				
**EO**	*Escherichia coli*	Φ mm = 20; MIC = 516 µg/ml	Algeria	Bahri et al. (2015)
	*Klebsiella pneumonia*	Φ mm = 29; MIC = 64,5 µg/ml		
	*Proteus mirabilis*	Φ mm = 20; MIC = 516 µg/ml		
	*Pseudomonas aeruginosa*	Φ mm = 20; MIC = 516 µg/ml		
	*Staphylococcus aureus*	Φ mm = 23; MIC = 268 µg/ml		
	*Candida albicans*	Φ mm = 22; MIC = 268 µg/ml		
**Aerial parts (Leaves and flowers)**				
**EO(S**_**1**_ **)**	*Staphylococcus aureus* (ATCC 6538)	Φ mm = 24.0 ± 4.2		
	*Staphylococcus aureus* (ATCC25923)	Φ mm = 38.5 ± 8.2		
	*Enterococcus faecalis* (ATCC13047)	Φ mm = 11.7 ± 0.6		
	*Escherichia coli* (ATCC 8739)	Φ mm = 10.0 ± 0.0		
	*Klebsiella pneumonia* (ATCC 700603)	Φ mm = 6.0 ± 0.0		
	*Pseudomonas aeruginosa* (ATCC 27853)	Φ mm = 6.0 ± 0.0		
	*Candida albicans* (ATCC 10231)	Φ mm = 12. ±1.2		
	*Candida albicans* (CIP 444)	Φ mm = 19.0 ± 1.0		
	*Aspergillus fumigatus* (MNHN 566)	Φ mm = 13.0 ± 1.7		
	*Aspergillus flavus* (MNHN 994294)	Φ mm = -		
**EO(S**_**2**_ **)**	*Staphylococcus aureus* (ATCC 6538)	Φ mm = 20.0 ± 1.7	Algeria	Boussaïd et al. (2022)
	*Staphylococcus aureus* (ATCC25923)	Φ mm = 24.0 ± 1.4		
	*Enterococcus faecalis* (ATCC13047)	Φ mm = 15.3 ± 1.2		
	*Escherichia coli* (ATCC 8739)	Φ mm = 11.3 ± 0.6		
	*Klebsiella pneumonia* (ATCC 700603)	Φ mm = 6.0 ± 0.0		
	*Pseudomonas aeruginosa* (ATCC 27853)	Φ mm = 6.0 ± 0.0		
	*Candida albicans* (ATCC 10231)	Φ mm = 15.0 ± 0.0		
	*Candida albicans* (CIP 444)	Φ mm = 11.3 ± 0.6		
	*Aspergillus fumigatus* (MNHN 566)	Φ mm = 8.3 ± 0.6		
	*Aspergillus flavus* (MNHN 994294)	Φ mm = 21.3 ± 4.2		
**EO(S**_**3**_ **)**	*Staphylococcus aureus* (ATCC 6538)	Φ mm = 20.0 ± 1.7		
	*Staphylococcus aureus* (ATCC25923)	Φ mm = 24.6 ± 0.9		
	*Enterococcus faecalis* (ATCC13047)	Φ mm = 9.3 ± 0.6		
	*Escherichia coli* (ATCC 8739)	Φ mm = 6.0 ± 0,0		
	*Klebsiella pneumonia* (ATCC 700603)	Φ mm = 6.0 ± 0.0		
	*Pseudomonas aeruginosa* (ATCC 27853)	Φ mm = 6.0 ± 0.0		
	*Candida albicans* (ATCC 10231)	Φ mm = 12.0 ± 0.0		
	*Candida albicans* (CIP 444)	Φ mm = 11.7 ± 1.2		
	*Aspergillus fumigatus* (MNHN 566)	Φ mm = 12.7 ± 2.1		
	*Aspergillus flavus* (MNHN 994294)	Φ mm = 20.0 ± 5.3		

**S**_**1**_: The sample was dominated by camphor (**33.7%**) as the major constituent; **S**_**2**_ **:** The sample was characterized by a high content of *α*-pinene (**50.2%**); **S**_**3**_: Bornyl acetate (**42.5%**) was the main compound in the sample.

^¥, γ, δ^ exhibited antagonism, synergism and partial synergism effects with Amox, respectively.

Achmit et al. (2021) tested the antibacterial activity of the essential oil of *T. articulata* sawdust from the root burl against nine strains of *Staphylococcus aureus* using the disc diffusion assay. The nine strains contained eight clinical isolates and one standard strain (ATCC 25923). They showed that the EO exhibited pronounced antibacterial properties against all *Staphylococcus aureus* tested strains, including two methicillin-resistant and one multidrug-resistant. The broth microdilution assay confirmed the high susceptibility of these strains to the EO, displaying MIC values ranging from 0.19 to 48 nL/ml and inhibition Zone Diameter (IZD) values ranging from 25 to 44 mm. The high inhibitory effects were ascribed to the significant amounts of monoterpenic phenols in the EO, such as carvacrol (37.8%). The antibacterial activity of *T. articulata* sawdust essential oil was corroborated by previous studies conducted by Talbaoui et al. (2016) and El Moussaouiti et al. (2010). Using disc diffusion assay, Talbaoui et al. (2016) demonstrated that the EO exhibited a strain-dependent inhibitory effect. *Escherichia coli* was the most sensitive to the EO, with MIC values of 5 μL/ml and inhibition zone diameter value of 19 mm, whereas *Enterococcus faecalis*, *Klebsiella pneumonia*, and *Streptococcus* D (Group D *Streptococcus*) were less susceptible to the EO. The authors identified thymol (22.83%) and 3-*tert*-butyl-4-methoxy phenol (35.02%) as the main compounds in the EO and correlated the effect with these key compounds or potential synergistic/antagonistic effects of all the mixture (Talbaoui et al., 2016). Likewise, *T. articulata* leaves essential oil demonstrated strong antibacterial effects towards *Escherichia coli*, *Bacillus subtilis*, *Staphylococcus aureus*, and *Micrococcus luteus*, with *Staphylococcus aureus* being the most vulnerable to the EO (Bourkhiss et al., 2007). In the same line, the antibacterial activity of *T. articulata* leaves essential oil was also assessed against five bacteria strains by Abi-Ayad et al. (2011) using the disc diffusion and the disc volatilization method. The EO inhibited the growth of *Bacillus cereus* (ATCC11778), *Staphylococcus aureus* (ATCC 25923), and *Escherichia coli* (ATCC 25922), with MIC values of 5, 7, and 10 μL/ml, and IZ values of 60.5, 22, and 19 mm, respectively. However, *Escherichia coli* (ATCC 25921) and *Pseudomonas aeruginosa* (ATCC 27853) have manifested resistance towards the EO, with an IZ value of 1 mm. In a recent study, Boussaïd et al. (2022) used the disc diffusion method to investigate the antimicrobial capacity of three samples obtained from *T. articulata* aerial parts (flowers and leaves) against three Gram-positive bacteria, three Gram-negative bacteria, two yeasts, and two filamentous fungi. Camphor (33.7%), *α*-pinene (50.2%), and bornyl acetate (42.5%) were the predominant compounds in sample 1, sample 2, and sample 3, respectively. Results suggested that all three samples were ineffective against the bacterial strains *Pseudomonas aeruginosa* (ATCC 27853) and *Klebsiella pneumonia* (ATCC 700603), while *Staphylococcus aureus* (ATCC 25923), *Candida albicans* (ATCC 10231), and *Aspergillus fumigatus* (MNHN 566) were the most susceptible to the EOs. On the other hand, the sample containing camphor as the dominant component was proven to be most prominent with MICs ranging between 1 and 3 μL/ml, whereas the essential oil enclosing *α*-pinene as the main ingredient was the least effective with MICs ranging between 2 and 6 μL/ml. Moreover, Djouahri et al. (2014) reported the potency of *T. articulata* extracts to increase the efficacy of the antibiotic Amox towards bacteria strains exhibiting the MDR phenotype. When they associated the antibiotic Amox with ETOH 100%, ETOH 70%, or water extracts of *T. articulata* fresh cones, they witnessed a synergism or partially synergism effect against *Staphylococcus aureus* (CIP 7625), *Salmonella enterica* (E32), *Klebsiella pneumoniae* (CIP 8291), *Escherichia coli* (ATTC 10536), *Listeria monocytogenes* (CIP 82110), and *Pseudomonas aeruginosa* (CIPA 22). However, when the antibiotic and MeOH 100% extract were combined, an antagonistic effect was noticed for all tested strains.

Previous studies revealed that *T. articulata* has strong *in vitro* antifungal effects. Ben Ghnaya et al. (2016) tested the antifungal potency of *T. articulata* leaves essential oil against phytopathogenic fungi, namely *Botrytis cinerea*, *Fusarium avenaceum*, *Fusarium oxysporum*, *Fusarium culmorum*, and *Fusarium solani* using *in vitro* contact tests. Results demonstrated that the essential oil exhibited significant mycelial growth inhibition against all the tested fungi. *Botrytis cinerea* was the most sensitive to the EO, with an inhibition percentage of 71.17%, whereas *Fusarium solani* was less susceptible to the EO (25.36%). In the context of investigating new eco-effective and environmentally friendly fungicides to neutralize the hazardous impacts of mycotoxins on the stored grains, Abi-Ayad et al. (2013) examined the antifungal properties of *T. articulata* leaves EO towards three fungal strains, namely *Aspergillus niger*, *Aspergillus flavus*, and *Fusarium* spp., through the contact method. The EO, at the following concentrations 5, 10, 15, and 20 μL/ml, exhibited significant inhibitory effects on the growth of the three fungi. After 6 days of incubation, the highest activity was shown against *Fusarium spp*., which was fully suppressed at a concentration of 20 µL/ml. In the same line, Bourkhiss et al. (2007) reported strong antifungal effects of *T. articulata* leaves essential oil against *Penicillium parasiticus* and *Aspergillus niger* at the concentration of 1/500 (V/V). The authors credited the significant antifungal capacity to the chemical profile of EO, which was dominated by bornyl acetate. Nevertheless, they did not rule out the possibility of a whole-compound synergism, which has yet to be validated. In spite of being endowed with strong antimicrobial properties, the majority of the assays were conducted using the disk diffusion test, which, while useful for evaluating the antimicrobial activity, may not always compare the potential of various extracts due to differences in physical features such a solubility, volatility, and diffusion in the in agar medium (Scorzoni et al., 2007). In contrast, the broth microdilution method is more accurate and gives better visualization of the inhibitory concentrations. Therefore, it is a feasible alternative for confirming the antibacterial efficacy of plant extracts. Furthermore, antibacterial research has largely been focused on plant crude extracts and essential oil, with no studies looking into the antimicrobial potential of isolated chemicals, particularly those recognized for their antimicrobial properties. Thus, in-depth studies incorporating *in vivo* and *in vitro* are recommended to offer a valid basis for investigating potential and low harmful antimicrobial compounds from the plant.

### Other activities

The *in vitro* antidiabetic activity of extracts from *T. articulata* was tested against α-amylase. Results revealed a dose-dependent inhibitory effect against α-amylase (IC_50_ of 57.74 μg/ml) following a competitive inhibition way (Toumi et al., 2022). However, further *in vitro* and *in vivo* studies are highly required to validate the ethnomedicinal use of the plant as a hypoglycemic agent. Moreover, the essential oil from *T. articulata* displayed moderate leishmanicidal potency against *L. infantum*, while no effect was recorded against *L. major* up to 8 μg/ml (Ahmed et al., 2011).

## Potential application of *T. articulata* essential oils as food preservatives

During the last decades, commercial food preservatives have extensively been applied to prevent spoilage and biodeterioration of processed foodstuffs, while retaining their natural flavors, taste, color, nutritional value, and appearance (Carocho et al., 2014). The severe defects linked to the employment of synthetic additives have fueled more and more controversies regarding their injurious effects on the behavior and health of consumers, including genotoxicity, carcinogenicity, asthma attacks, eczema, skin rashes, and nervousness, among others (Carocho et al., 2014; Amchova et al., 2015; Pandey et al., 2021). On the other hand, widespread distrust of synthetic additives, especially in developed countries, has driven scientists toward seeking new natural-based and health-beneficial preservatives with pronounced antimicrobial and antioxidant properties (Pandey et al., 2021). Indeed, a myriad of plant species from the Cupressaceae family are distinguished by high amounts of volatiles components, which could serve as a renewable supply of food additive agents due to their substantial cidal/static action (Benjemaa et al., 2022; Sadiki et al., 2022). *T. articulata* essential oil is almost colorless or pale yellow, featured by its sweet balsamic scent with a broad spectrum of antimicrobial and antioxidant properties (Djouahri et al., 2016; Achmit et al., 2021; Boussaïd et al., 2022; Sadiki et al., 2022). In a recent study, the effect of *T. articulata* EO on the shelf life of refrigerated storage chicken fillets was explored by Salem et al. (2022). Results disclosed the capacity of *T. articulata* EO at the concentration of 200 ppm/100 g of product to drastically drop lipid oxidation (*p* < 0.05) over 12 days of refrigerated storage. Interestingly, *T. articulata* EO showed significant antioxidant activity (IC_50_ = 1,000 μg/ml) with no toxicity on murine macrophage cells and the ability to reduce the acidity of the treated fillets (1.3 g/kg) from the third day of storage compared with the control. Results also revealed the capacity of *T. articulata* EO to significantly decrease the flora charges and inhibit the growth of food-borne pathogen bacteria strain *Enterococcus faecalis* (ATCC 29212) with MIC <0.031 mg/ml (Salem et al., 2022). Thereby, *T. articulata* EO could be a potential source of chemical compounds that might be used to prevent biodeterioration and spoiling of perishable foods such as meat and poultry during storage time.

## Toxicity studies

It is compulsory to evaluate the safety of *T. articulata* as one of the most frequently used medicinal plants in North Africa countries. El Jemli et al. (2016) examined the acute toxicity of the leaves EO at doses of 2 and 5 g/kg after oral administration to Swiss mice. For the first 6 h and within the next 2 weeks following the administration of the EO, animals were examined for bodyweight fluctuations, mortality, neurotoxicity, and convulsions signs. As a result, the administration of *T. articulata* EO at both doses (2 and 5 g/kg) showed relatively low acute toxicity and did not cause mortality. Interestingly, at the dose of 2 g/kg, there was no change in animals’ weight, growth, or functions as compared to the control group. However, at 5 g/kg, a substantial drop in body weight and activities were recorded, which is likely related to a neurotoxic effect. Moreover, Rachid et al. (2018) have also demonstrated that the crude aqueous extract of the leaves of *T. articulata* did not exhibit cytotoxicity towards PLP 2 (porcine liver cells), with an IC_50_ higher than 400 μg/ml, compared to Ellipticine, which has an IC_50_ of 3.2 μg/ml. It is worth noting that the crude extract in this study was highly selective for cancer cell lines and had no effect on non-tumor cells PLP2. Nevertheless, for plant applications and novel drugs development, toxicology safety evaluation is mandatory. On the other hand, toxicological investigations of extracts and components obtained from *T. articulata* have yet to be completely addressed. As a result, more toxicity studies are required to establish the appropriateness of the plant extracts and related bioactive constituents.

## T*. articulata* extracts as a botanical pesticide for a sustainable agriculture

Botanical pesticides are naturally occurring agents derived from plants of different families, which are applied similarly to chemical pesticides as plant extracts, essential oils, or both to achieve pest management in an eco-friendly way (Ukoroije and Otayor, 2020; Bitchagno et al., 2022). Biopesticides would account for nearly 20% of the worldwide pesticide market by 2025 compared to 4–5% in 2014 (Isman, 2015). Due to the harmful effects associated with the use of synthetic pesticides on human health and biodiversity, the recent trend is being shifted towards plant-based pesticides. Indeed, several plant families, including Apiaceae, Asteraceae, Cupressaceae, Lamiaceae, and Rutaceae, have been proven to exhibit promising pesticidal properties due to the presence of a broad spectrum of secondary metabolites, such as alkaloids, phenols, flavonoids, steroids, and tannins, among others (Lengai et al., 2020). To make plant-based pesticide, various parts from these plants, such as leaves, stems, bark, roots, rhizome, are dried, powdered then subjected to extraction with the suitable solvent to maximize the yield of the target bioactive compounds, which are then concentrated, formulated, and evaluated for their safety (Duke et al., 2010; Lengai et al., 2020; Ukoroije and Otayor, 2020). In fact, several plant-based pesticides have been successfully formulated and commercialized as safe pesticides for crop pest management, such as pyrethroids derived from the dried flowers of *Tanacetum cinerariaefolium* and azadiractin from *Azadirachta indica*. Botanical pesticides have a variety of mechanisms of action on pests, including repellence, molluscicides, attractants, nematicides, and phytotoxins (Ramdani et al., 2021; Souto et al., 2021). Botanical pesticides, unlike conventional pesticides, are made up of several bioactive components rather than a single active agent. Thus, they may affect both physiological and behavioral processes owing to their internal synergism or antagonism (Miresmailli and Isman, 2014; Niroumand et al., 2016). Consequently, it is unlikely for pests to develop resistance to such a mixture of natural pesticidal agents (Niroumand et al., 2016). For example, Pyrethrums disrupt the normal transmission of nerve impulses by altering the sodium and potassium ion exchange process in insect nerve fibers, which result in paralysis and death (neurotoxicity) (Laxmishree and Singh, 2018). Botanical pesticides may also inhibit digestive enzymes, such as amylase, invertase, lipase, and protease. They can affect the rate of enzyme-substrate complex breakdown by decreasing enzyme affinity to the substrate and increasing Km (Zibaee, 2011; Sengottayan, 2013).

To efficiently and sustainably control *Aphis citricola*, a citrus crop pest, while minimizing the reliance on hazardous synthetic pesticides. Harmouzi et al. (2016) investigated *T. articulata* EO chemical composition and tested its toxicity on *A. citricola*. They showed that EO exhibited significant acute toxicity against *A. citricola* by contact in a dose and time-dependent fashion (Table 7). The authors recorded an LC_50_ of 54.03 μL/L after 24 h of exposure. They attributed the significant toxicity to the significant amounts of oxygenated monoterpenes like isobornyl acetate, borneol, and camphor, which can interfere with several neurotransmitters such as Octopamine and causes neurotoxicity. To come up with new effective eco-friendly methods to control mosquitoes responsible for Vector-borne diseases, Aouinty et al. (2006) assessed the larvicidal potency of *T. articulata* wood aqueous extract against four mosquito species. Results showed that the extract exhibited a significant larvicidal effect against all mosquitoes’ larvae at a concentration of 4%, with a mortality rate of 100% after 24 h of exposure. *Botrytis cinerea* is a necrotrophic fungus that causes gray mold in tomatoes and other crops such as peppers, cabbage, beans, etc. To seek safe, efficacious, and natural fungicides, Rguez et al. (2020) assessed the *in vivo* and *in vitro* antifungal properties of *T. articulata* essential oil at different phenological stages against *B. cinerea*. The authors reported that *T. articulata* EO exhibited the highest antifungal activity at the blooming phase. They stated that *T. articulata* EO applied as a means of prevention on tomato plants under greenhouse conditions improved plant growth and decreased *B. cinerea* infection to 17.72% compared to 52.1% documented in non-treated plants. When they applied 100 μg/ml of EO on tomato fruit, they observed that the infection rate recedes by 64.01%. They associated the antifungal effect with the presence of oxygenated monoterpenes and their hydrocarbons and supported the possible application of *T. articulata* EO as a natural fungicide.

**TABLE 7:** Biocidal properties of *T. articulata*-based extracts.

Extracts	Pests controlled	Mode of preparation and application	Key results	References
EO (at different phenological stages)	*Botrytis cinerea*	The pulverization of essential oil as preventive treatment on detached tomato leaflets	The EO at the concentration of 2 mg/ml prevents symptoms of alteration and reduces the infection of *B. cinerea* between 54.90 and 64.30%	Rguez et al. (2020)
EO	*Aphis citricola*	The EO was applied topically on the dorsal thorax of aphids	*T. articulata* leafy twigs EO exhibited time and concentration-dependant repulsive activity against *A. citricola*	Harmouzi et al. (2016)
MeOH extract	*Sitophilus oryzae L*	The insecticidal effect was assessed using contact toxicity (Whatman paper was impregnated with different doses, then placed in a Petri dish with 20 insects)	The extract was highly toxic and caused mortality of 95.65% after 24 h at a dose of 60 mg/cm^2^	Dane et al. (2016)
Wood aqueous extract	*Daphnia magna*	Acute and reprotoxicity of the aqueous extracts (from 0.5 mg/L to 17 mg/L) was assessed on freshwater cladoceran *Daphnia magna* using immobilization as an endpoint	The extract at various concentrations exhibited significant acute toxicity and reprotoxicity on *D. magna* population	Montassir et al. (2017)

## Patents related to *T. articulata* published between 2001 and 2022

Recently, there has been a global cumulative increase in the number of *T. articulata*-related patents, demonstrating the intensive ongoing research on this plant tree to provide high-value-added products and support its usage to treat various health conditions. As reported in (Table 8) various parts from the plant, especially resin, have been formulated as patents and found applications mainly in the pharmaceutical field to prevent and treat neurodegenerative diseases such as Alzheimer’s disease, diabetes mellitus, fibrotic conditions, cancers, among others. These patents have the specificity to combine cutting-edge extraction methods with tried-and-true formulas to ensure both efficacy and safety. For instance, a *cis*- and *trans*-communic acid rich-fraction obtained from *T. articulata* resin using a two-step or three-step extraction procedure (with a carrier) was designed to prevent and treat fibrotic conditions, gliosis, surgical adhesions, and impaired neurological function such as vascular dementia and Alzheimer’s disease (Hazan and Lucassen, 2015). Moreover, an herbal formulation consisting of several plant genera, including *T. articulata*, displayed the capacity to prevent and treat diabetes mellitus and its related health complications and dyslipidemia (Fogel, 2010). The usefulness of *T. articulata* in cancer prevention and treatment has been alarmed by a couple of inventions (Brooks and Norris, 2007). The first patent was invented by Brooks and Norris, (2007), claiming the use of Pheophorbide derivative compounds from plants as potential agents (high oral bioavailability) to inhibit cell proliferation and angiogenesis. Another patent invented by Benoit, (2006) furnished a method for the extraction and purification of potential compounds from various plants, including *T. articulata*, having the ability to inhibit, slow down, or prevent cell migration of abnormal cells. In the cosmetic field, Mitsuharu and Michimasa (2001) developed a cosmetic formulation with the steam distillate of *T. articulata*, which aimed at providing dry skin gloss and tension. Several formulations from plant extracts, including *T. articulata*, claimed the capacity to prevent and treat various dermatological conditions, including skin sagging, irradiation-induced skin damage, skin lines, and elastotic changes in the skin. The dermatological action of the plant extracts seemed to be related to the inhibition of one or more extracellular proteases such as Matrix metalloproteinase-1 (MMP-1), Matrix metalloproteinase-2 (MMP-2), and human leukocyte elastase (HLE). Other patents included the one issued in 2019 by Toni, which claimed the use of *T. articulata* sandarac and beeswax to manufacture a self-healing cutting board or a storage container characterized by their water-resistant, non-skid, and antimicrobial properties (Toni, 2019). Further details about patents granted for therapeutic and cosmetic applications of *T. articulata* are reported in Table 8.

**TABLE 8:** Patents related to *T. articulata* published between 2001 and 2022.

Patent number	Title	Issue date	Description of the invention	References
US11357246	High intensity sweeteners	2022-01-14	The patent relates to the fields of foods, chemistry, beverages, and other edible components. It focuses on sweet-tasting compounds, sweet taste enhancers, and combinations thereof for ingestible compositions such as beverages, foods, as well as other orally delivered therapeutic products	Patron et al. (2022)
JP2021104190	Space adjustment device and space adjustment method	2021-07-26	A space-adjusting device comprising 14 medicinal plants releases volatile components acting on a *γ*-aminobutyric acidergic (GABA) nerve receptor in olfaction to relieve mental stress in narrow spaces like offices	Takashi, (2021)
US10337139	Combination of an organic substrate and organic formulation for use as a cutting board and storage container	2019-07-02	A formulation consisting of bees wax and *T. articulata* sandarac was invented to serve as a self-healing cutting board or a storage container. The board and the storage containers are water resistant, non-skid antimicrobial, re-useable and compostable	Toni, (2019)
US20170143022	Compositions Incorporating an Umami Flavor Agent	2017-04-25	The present invention relates to formulations consisting of an Umami flavor agent in combination with one or more other food additives, suitably derived from herb, spice, fat, or oil to improve sweeteners, bittering agents, acid/sour flavor agents, or salts	Wicker and Ditschun, (2017)
US20170096418	Compounds useful as modulators of TRPM8	2017-04-06	The present invention relates to compounds beneficial as cooling agents, which are capable of modulating Melastatin Transient Receptor Potential Channel 8 (TRPM8) involved in the chemesthetic sensation to generate a cooling effect	Patron et al. (2017)
US20150246087	Extracts and therapeutic uses thereof	2015-09-03	A *cis*- and *trans*-communic acid rich-fraction retrieved from *T. articulata* resin using a two-step or three-step extraction procedure (with a carrier) aimed at preventing and treating fibrotic conditions, gliosis, surgical adhesions, and impaired neurological function such as vascular dementia and Alzheimer’s disease	Hazan and Lucassen, (2015)
US20110311661	Plant extracts and dermatological uses thereof	2011-12-22	The invention relates to dermatology, wherein dermatological formulations from plant extracts (at least one plant, including *T. articulata*) are designed to treat different dermatological conditions, including skin sagging, irradiation-induced skin damage, and skin lines	Behr et al. (2011)
US20100292193	Radioprotective drugs	2010-11-18	The invention relates to a method consisting of the administration of cyclopiazonic acid (CPA) and/or a cyclopiazonic acid derivative to a subject to reduce radiation damage to tissues or cells during radiotherapy	McBride et al. (2010)
US20070299046	Orally available light-independent antineoplastic compounds	2007-12-27	The patent claims the capacity of the pheophorbide derivative compounds from plants to inhibit cell proliferation and angiogenesis	(Brooks and Norris, 2007)
US20060228426	Plant extracts for treatment of angiogenesis and metastasis	2006-10-12	The invention relates to modulators of cellular activity, which consists of plant extracts from several plants, including T. articulata, capable of inhibiting, slowing down, or preventing cell migration such as neoplastic cells	Benoit, (2006)
JP2001220312	Cosmetic composition containing steam distillate of plant	2001-08-14	The invention relates to the cosmetic field. Indeed, several plants from different families, including *T. articulata* from the Cupressaceae, were steam distilled to obtain a new safe cosmetic formula that is used to provide dry skin tension and shine	(Mitsuharu and Michimasa, 2001)

## Conclusion and perspectives

*T. articulata* has long been exploited for therapeutic, artistic, and ritual purposes. Recent ethnobotanical surveys reported the use of different parts, such as leaves, resin, bark, and cones, to cure several pathological conditions, including chronic ones such as diabetes mellitus and hypertension. According to several phytochemical studies, the plant extracts contain significant amounts of phenolic compounds, which are primarily made up of phenolic acids, flavonoids and their derivatives. Phenolic compounds, especially, flavonoids are excellent free radical scavengers. They may play a crucial role in mitigating and protecting the human body from free radicals-related diseases such as diabetes mellitus, cancers, and neurodegenerative diseases. As such, they have strongly been associated with the significant cytotoxic, antioxidant, anti-Alzheimer, and anti-inflammatory properties of *T. articulata* extracts.

Despites rich literature about the plant, the chemistry and biology of *T. articulata* have yet to be thoroughly examined. For instance, the phytochemical screening of *T. articulata* cones methanolic extract has positively identified other classes of compounds such as alkaloids and saponosides (Saber et al., 2021). Likewise, the individual compounds and their pharmacological effects have yet to be determined.

*T. articulata* has long been used by indigenous people in Morocco, Algeria, and Tunisia to treat digestive and respiratory disorders, cough, and diarrhea. These empirical practices were the springboard that has incentivized further antimicrobial studies over the last 2 decades. Recent phytochemical investigations of the essential oil revealed the presence of monoterpene hydrocarbons, oxygenated monoterpenes, sesquiterpene hydrocarbons, and oxygenated sesquiterpene. These chemical compounds have strongly been linked to the significant antimicrobial potency of the essential oil towards Gram-negative and Gram-positive bacteria exhibiting multidrug resistance phenotype (MDR). Interestingly, a synergistic effect has been recorded between the plant extracts (EtOH 70%, EtOH 100%), and the antibiotic Amoxicillin, improving the effectiveness of the antimicrobial agent.

Although this plant has long been used to manage a variety of chronic diseases such as diabetes mellitus and hypertension, limited pharmacological research has been conducted to corroborate and support these ethnomedicinal claims with scientific evidence. Therefore, further *in vitro* studies are highly required to evaluate the antidiabetic properties of the *T. articulata*-based extracts against *α*-amylase, *α*-glucosidase, and *β*-glucosidase. *In vivo* studies of the plant extracts in normal and streptozotocin (or alloxan)-induced diabetic rats are also mandatory to assess the extracts’ ability to lower blood glucose levels, restore the body cells’ sensitivity, and protect pancreatic cells. *T. articulata* extracts have also demonstrated potent *in vitro* cytotoxic effects with high selectivity towards various cancer cell lines. However, in-depth *in vivo* studies are also needed to explore the ability of the extracts to induce apoptosis in cancer cells through the intrinsic and extrinsic pathways. Further *in vivo* toxicological and clinical studies are required to ensure the plant’s efficacy and safety. In line with the traditional uses of the plant, *in vitro* and *in vivo* studies about the claimed anti-asthmatic, anti-tuberculosis, and wound healing properties are also mandatory. Likewise, locals in North Africa have also utilized *T. articulata* tar to cure parasitic illnesses, scabies, and inflamed wounds in livestock. Nevertheless, no anthelmintic studies have been conducted to back up these claims and provide scientific evidence for ethnoveterinary applications. The EO from the plant disclosed also the capacity to extend the shelf life of refrigerated storage chicken fillets, which indicates that the EO might be a source of chemical compounds that can be utilized as natural food additives in the food industry and as a platform for biodegradable active packaging.

## Abbreviations

A549, Human lung cancer cell line; A64-CLS, Cellosaurus cell line; ABTS, 2,2′-Azino-bis(3-ethylbenzothiazoline-6-sulfonic acid); DPPH, 2,2-Diphenyl-1-picrylhydrazyl; EO:, Essential oil; FRAP, Ferric reducing antioxidant power; HeLa, Cervical epithelial carcinom; HepG2, Liver hepatocellular carcinoma; HT29, Colon cancer cell line; IUCN, International Union for Conservation of Nature; IZD, Inhibition Zone Diameter; MBC, Minimum Bactericidal Concentration; MCF-7, Human breast cancer cell line; MDR, Multidrug resistance; MeOH, Methanolic extract; MeWo, Human melanoma cell line; MIC, Minimum inhibitory concentration; NCI-H460, Human Lung Carcinoma; PC-3, Human Prostate Adenocarcinoma; Sk-Br-3, Human breast cancer; Sk-OV-3, Human ovarian cancer; T24, Urinary bladder carcinoma; UACC-62, Melanoma cell line; edw, equivalent dry weight.
